# The CK1ε/SIAH1 axis regulates AXIN1 stability in colorectal cancer cells

**DOI:** 10.1002/1878-0261.13624

**Published:** 2024-02-28

**Authors:** Mengfang Yan, Zijie Su, Xiaoyi Pang, Hanbin Wang, Han Dai, Jiong Ning, Shanshan Liu, Qi Sun, Jiaxing Song, Xibao Zhao, Desheng Lu

**Affiliations:** ^1^ Guangdong Provincial Key Laboratory of Regional Immunity and Disease, International Cancer Center, Marshall Laboratory of Biomedical Engineering, Department of Pharmacology Shenzhen University Medical School, Shenzhen University China; ^2^ School of Pharmacy Shenzhen University Medical School, Shenzhen University China; ^3^ Department of Research The Affiliated Tumor Hospital of Guangxi Medical University Nanning China; ^4^ Medical Scientific Research Center, Life Sciences Institute Guangxi Medical University Nanning China

**Keywords:** AXIN1, CK1ε, colorectal cancer, SIAH1, ubiquitination, Wnt/β‐catenin

## Abstract

Casein kinase 1ε (CK1ε) and axis inhibitor 1 (AXIN1) are crucial components of the β‐catenin destruction complex in canonical Wnt signaling. CK1ε has been shown to interact with AXIN1, but its physiological function and role in tumorigenesis remain unknown. In this study, we found that CK1δ/ε inhibitors significantly enhanced AXIN1 protein level in colorectal cancer (CRC) cells through targeting CK1ε. Mechanistically, CK1ε promoted AXIN1 degradation by the ubiquitin–proteasome pathway by promoting the interaction of E3 ubiquitin‐protein ligase SIAH1 with AXIN1. Genetic or pharmacological inhibition of CK1ε and knockdown of *SIAH1* downregulated the expression of Wnt/β‐catenin‐dependent genes, suppressed the viability of CRC cells, and restrained tumorigenesis and progression of CRC *in vitro* and *in vivo*. In summary, our results demonstrate that CK1ε exerted its oncogenic role in CRC occurrence and progression by regulating the stability of AXIN1. These findings reveal a novel mechanism by which CK1ε regulates the Wnt/β‐catenin signaling pathway and highlight the therapeutic potential of targeting the CK1ε/SIAH1 axis in CRC.

## Graphical abstract

This study found that CK1δ/ε inhibitors stabilized the AXIN1 protein through targeting protein kinase CK1ε. CK1ε enhanced AXIN1 degradation by the ubiquitin–proteasome pathway by promoting the interaction of E3 ubiquitin‐protein ligase SIAH1 with AXIN1 in colorectal cancer cells. These findings reveal a novel mechanism underlying the roles of CK1ε in Wnt/β‐catenin signaling and colorectal carcinogenesis.

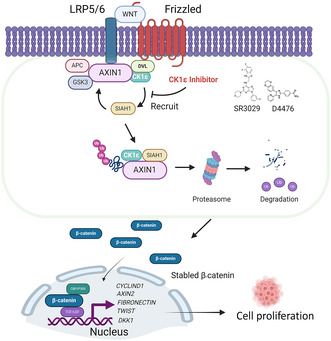

**Abbreviations**
APC: adenomatous polyposis coli
AXIN1: axis inhibitor 1
CHX: cycloheximide
CK1: casein kinase 1
CK1ε: casein kinase 1ε
CK1ε‐KO: *CSNK1E*‐deificient
CRC: colorectal cancer
DVL: disheveled
GSK3β: glycogen synthase kinase 3β
MID: MEKK1‐interacting domain
NSCLC: non‐small cell lung cancer
SIAH1: E3 ubiquitin ligase seven in absentia homolog 1
Wnt3a‐CM: Wnt3a conditioned medium
WT: wild‐type
λ‐PPase: λ‐phosphatase

## 1 Introduction

Colorectal cancer (CRC) is one of the most common malignancies worldwide [1]. Multiple signaling pathways, including p53 signaling [2], Notch signaling [3, 4], Hedgehog signaling [5, 6], and Wnt/β‐catenin signaling [7, 8], have been involved in the tumorigenesis of CRC. Moreover, at least 90% of CRC patients have mutations in genes encoding Wnt/β‐catenin signaling components, such as *APC* (73%) and *CTNNB1* (5%) [1, 9]. These critical components of the Wnt/β‐catenin signaling pathway are thought to be promising therapeutic targets for CRC [10]. However, the development of targeted therapies against these components in Wnt/β‐catenin signaling remains a significant challenge [11]. Therefore, understanding the molecular mechanisms of Wnt/β‐catenin signaling is essential to developing novel therapies for CRC.

The Wnt/β‐catenin signaling pathway is a precisely regulated and evolutionarily highly conserved signaling pathway. Aberrant activation of the Wnt/β‐catenin signaling pathway is widely associated with a variety of human diseases such as Alzheimer's disease, heart disease, osteoarthritis, and cancer [12]. The activation of Wnt/β‐catenin signaling is dependent on the level and subcellular location of β‐catenin. The level of β‐catenin is regulated by the destruction complex, which is composed of components such as axis inhibitor 1 (AXIN1), glycogen synthase kinase 3β (GSK3β), casein kinase 1 (CK1), and adenomatous polyposis coli (APC).

The AXIN1 acts as a scaffolding protein that binds directly to key components of the β‐catenin destruction complex, a negative regulator of the canonical Wnt signaling pathway [13]. Thus, modulation of AXIN1 plays a crucial role in the Wnt signaling pathway. AXIN1 is subject to a wide variety of post‐translational modifications, including sumoylation, ubiquitination, phosphorylation, and ADP ribosylation [14, 15, 16, 17, 18]. Previous studies reported that tankyrase inhibitor XAV939 stabilized AXIN1 protein by suppressing tankyrase‐RNF146 axis‐mediated AXIN1 ubiquitination and degradation [18, 19, 20]. USP34 and USP7 have also been showed to bind to AXIN1 and stabilize AXIN1 protein to regulate Wnt signaling [21, 22]. Furthermore, E3 ubiquitin ligase seven in absentia homolog 1 (SIAH1) has been found to be involved in the Wnt‐induced degradation of AXIN1 by binding to the GSK3β‐binding domain of AXIN1 [23]. However, there is still lack a clear understanding of the molecular mechanism by which the activation of the Wnt signaling pathway induced AXIN1 degradation [24, 25].

Casein kinase 1 (CK1), is an evolutionarily conserved serine/threonine‐specific protein kinase. CK1 family members play an essential role in membrane transport, cell division, DNA repair, and circadian rhythms as well as Wnt and Hedgehog signaling pathways [26, 27, 28]. As a key molecule in the Wnt signaling pathway, CK1 family members phosphorylate several crucial components of the Wnt signaling pathway. In the presence of Wnt ligands, CK1γ binds to the membrane via C‐terminal S‐palmitoylation and phosphorylates LRP5/6 at Thr1479, exposing a docking site for AXIN1 [29, 30]. Additional two isoforms of the CK1 kinase family, CK1δ/ε, are also able to phosphorylate LRP5/6 and facilitate the recruitment of AXIN in β‐catenin destruction complex to the plasma membrane, which causes the disassembly of the destruction complex, leading to β‐catenin accumulation and nuclear translocation [30]. Moreover, CK1δ/ε phosphorylates APC in the presence of GSK3β, which enhances the affinity of APC for β‐catenin, causing β‐catenin to dissociate from the destruction complex and bind to APC, promoting β‐catenin interaction with the E3 ubiquitin ligase β‐Trcp [31, 32]. In addition, the cytoplasmic adaptor protein disheveled (DVL) is phosphorylated by CK1ε via directly binding to the DEP and PDZ domains of DVL, thereby disrupting β‐catenin destruction complex [27]. In the absence of Wnt ligands, CK1α and GSK3β sequentially phosphorylate β‐catenin [33, 34], which results in β‐catenin binding to β‐Trcp and proteasome degradation [35]. It is now well established from a variety of studies that CK1δ/ε could bind to AXIN1, but the functional significance of this binding is unclear [36].

Currently, several small molecule CK1δ/ε inhibitors have been developed to achieve beneficial effects in the treatment of different tumor entities, including CKI‐7 [37], D4476 [38, 39], PF670462 [40, 41], IC261 [26], and SR3029 [42]. CK1 family members might serve as a potent therapeutic target for cancers. Rosenberg et al. [43] reported that SR3029 inhibited the growth of breast cancer *in vitro* and *in vivo*, showing a good promise to be an effective antitumor agent against breast cancer. It is urgent to deeply understand the molecular mechanism underlying the actions of CK1δ/ε inhibitors for the development of effective therapeutics.

In this study, we observed that the CK1δ/ε inhibitors significantly enhanced the stability of AXIN1 protein through targeting CK1ε. We further explored the mechanism by which CK1ε promoted AXIN1 degradation. Our results identified a CK1ε‐SIAH1 axis for the regulation of AXIN1 stability, and this axis is implicated in colorectal carcignogenesis.

## 2 Materials and methods

### 2.1 Reagents and plasmids

SR3029, MG132, Bafilomycin A1 (Baf‐A1) were purchased from MedChemExpress (Shanghai, China). Cycloheximide (CHX) was purchased from Merck (Darmstadt, Germany). Chloroquine (CQ) was purchased from Selleck Chemicals (Shanghai, China). The following primary antibodies were used: anti‐CK1ε (AB270997) and anti‐SIAH1 (AB2237) were purchased from Abcam (Boston, MA, USA). Anti‐AXIN1 (2087S), anti‐HA (33724S), anti‐Ubiquitin (58395S), IgG (5415S), anti‐V5 (13202S), and anti‐CyclinD1 (2978S) were purchased from Cell Signaling Technology (Boston, MA, USA). Anti‐GAPDH (6004‐1‐Ig), anti‐GFP (66002‐1‐Ig), anti‐Flag (20543‐1‐AP), and anti‐β‐Actin (81115‐1‐RR) were purchased from Proteintech (Wuhan, China). The expression plasmids encoding Flag/V5/GFP‐tagged AXIN1 and HA‐tagged SIAH1 were constructed using standard molecular biology techniques. The expression vectors for Myc/HA‐tagged Ub were purchased from WZ Biosciences (Jinan, China). The expression plasmids for pCMXβgal and GFP/V5‐tagged CK1ε have been described previously [44]. The SuperTOPFlash reporter plasmid was provided by Karl Willert (University of California at San Diego, La Jolla, CA, USA).

### 2.2 Cell culture

HEK293T (RRID: CVCL_0063) and SW480 (RRID: CVCL_0546) cells were cultured in DMEM containing 10% fetal bovine serum, while HT29 (RRID: CVCL_A8EZ), HCT116 (RRID: CVCL_0291) and MC38 (RRID: CVCL_0A68) cells were cultured in RPMI1640 with 10% fetal bovine serum. HEK293T, SW480, HT29, HCT116, MC38 cells were obtained from American Type Culture Collection (ATCC, Manassas, VA, USA). The *CSNK1E*‐knockout (KO) HEK293T cell line was constructed using standard protocol. All cell cultures were maintained at 37 °C in 5% CO_2_ and 95% humidity in the appropriate media. All cell lines have been authenticated by short tandem repeat (STR) analysis in the past 3 years. Besides, all experiments were carried out with mycoplasma‐free cells, routinely tested by mycoplasma PCR detection kit (Beyotime, Shanghai, China).

### 2.3 Generation of *CSNK1E*‐knockout cells

The *CSNK1E*‐KO variants of the HEK293T cells were generated by CRISPR/Cas9. The guide RNA sequence (5′‐GCAGGTGCCAACATCGCCTC‐3′) was obtained from the CHOPCHOP website (https://chopchop.cbu.uib.no/).

### 2.4 Lentiviral shRNA

The sequences of CK1ε, SIAH1 (Human), Siah1α (Mouse) shRNAs were as follows: shCK1ε‐1: sense CCGGCCAGTGTTTGCTTAGTGTCTTCTCGAGAAGACACTAAGCAAACACTGGTTTTTG, anti‐sense AATTCAAAAACCAGTGTTTGCTTAGTGTCTTCTCGAGAAGACACTAAGCAAACACTGG; shCK1ε‐2: sense CCGGCTCTTACCTACGTCAGCTCTTCTCGAGAAGAGCTGACGTAGGTAAGAGTTTTTG, anti‐sense AATTCAAAAACTCTTACCTACGTCAGCTCTTCTCGAGAAGAGCTGACGTAGGTAAGAG; and shSIAH1: sense CCGGGAAGCGACTCCTCGATCTATTCTCGAGAATAGATCGAGGAGTCGCTTCTTTTTG, anti‐sense AATTCAAAAAGAAGCGACTCCTCGATCTATTCTCGAGAATAGATCGAGGAGTCGCTTC, shSiah1α: sense CCGGGCATCAGCACAAGTCCATTACCTCGAGGTAATGGACTTGTGCTGATGCTTTTTG, anti‐sense AATTCAAAAAGCATCAGCACAAGTCCATTACCTCGAGGTAATGGACTTGTGCTGATGC. For infection with lentivirus, the cells were cultured with lentiviral solution for 24 h in the presence of 4 μg·mL^−1^ polybrene (Merck, Darmstadt, Germany).

### 2.5 Luciferase reporter assays

HEK293T cells were seeded in 24‐well plates and co‐transfected with SuperTOPFlash reporter plasmid, pCMXβgal (for normalizing the transfection efficiency) and the indicated expression plasmids. The cells were harvested after 48 h. Luciferase assay was performed using a dual‐specific luciferase assay kit (Promega, Madison, WI, USA) according to the manufacturer's instructions.

### 2.6 Co‐immunoprecipitation

Cells were washed with PBS and lysed with RIPA buffer (50 mm Tris–HCl pH7.4, 150 mm NaCl, 1% NP‐40, 0.1% SDS, containing phosphatase‐inhibitor cocktail, protease inhibitor cocktail and 1 mm PMSF) followed by mild sonication on ice, the cell lysates were centrifuged at 13 400 ***g*** for 15 min, the supernatant were incubated with appropriate antibodies or antibody‐conjugated beads overnight at 4 °C.

### 2.7 Real‐time polymerase chain reaction analyses

Total RNA was extracted from cells or tumor tissues with RNAiso Plus (Takara, Kusatsu, Japan), and reverse‐transcribed into cDNA using Primescript RT Reagent Kit (Takara) according to the manufacturer's instructions. Gene expression was examined using Eastep qPCR Master Mix (Promega), and gene expression was normalized to *GAPDH* using the ΔΔ*C*_t_ method. The gene‐specific primers were as follows: *HsAXIN1* sense, 5′‐GGTTTCCCCTTGGACCTCG‐3′ and anti‐sense, 5′‐CCGTCGAAGTCTCACCTTTAATG‐3′; *HsAXIN2* sense, 5′‐CAACACCAGGCGGAACGAA‐3′ and anti‐sense, 5′‐GCCCAATAAGGAGTGTAAGGACT‐3′; *HsCYCLIND1* sense, 5′‐GGCGGAGGAGAACAAACAGA‐3′ and anti‐sense, 5′‐TGGCACAAGAGGCAACGA‐3′; *HsDKK1* sense, 5′‐GGAATAAGTACCAGACCATTGACAAC‐3′, and anti‐sense, 5′‐GGGACTAGCGCAGTACTCATCA‐3′; *HsTWIST* sense, 5′‐GGCTCAGCTACGCCTTCTC‐3′ and anti‐sense, 5′‐TCCTTCTCTGGAAACAATGACA‐3′; *HsFIBRONECTIN* sense, 5′‐ACCTACGGATGACTCGTGCTTT‐3′, and anti‐sense, 5′‐TTCAGACATTCGTTCCCACTCA‐3′; *HsGAPDH* sense, 5′‐TGTGGGCATCAATGGATTTGG‐3′ and anti‐sense, 5′‐ACACCATGTATTCCGGGTCAAT‐3′; *mMGapdh* sense, 5′‐AGGTCGGTGTGAACGGATTTG‐3′ and anti‐sense, 5′‐TGTAGACCATGTAGTTGAGGTCA‐3′; *mMCyclinD1* sense, 5′‐CCACCTGCAAGACCATCGAC‐3′ and anti‐sense, 5′‐CTGGCGAGCCTTAGTTTGGA‐3′, *mMAxin2* sense, 5′‐AACCTATGCCCGTTTCCTCTA‐3′ and anti‐sense, 5′‐GAGTGTAAAGACTTGGTCCACC‐3′.

### 2.8 Colony formation assays

Cells were seeded in 6‐well plates with 500 cells per well, then incubated until a macroscopic cell mass was formed (the medium was changed every 3 days), after about 2 weeks, the cells were stained with crystal violet and photographed.

### 2.9 Cell viability assays

Cells were seeded in 96‐well plates (3 × 10^3^ cells per well), 10 μL of 5 mg·mL^−1^ MTT solution was added to the cells after 24, 48 or 72 h, incubated at 37 °C for 4–6 h, 100 μL DMSO was added, mixed and absorbance was measured at 570 nm.

### 2.10 Immunofluorescence analyses

HEK293T cells were co‐transfected with various expression plasmids, then fixed with 4% paraformaldehyde, permeabilized with 0.5% Triton X‐100, and the slides were blocked with 10% goat serum, following incubation with the primary antibody at 4 °C overnight. The next day, the slides were incubated with the secondary antibody (Alexa Fluor 488‐conjugated anti‐mouse antibody and Alexa Fluor 594‐conjugated anti‐rabbit antibody, Life Technologies, Carlsbad, CA, USA) for 1 h, then the nuclei were stained with DAPI, sealed and photographed using a fluorescence microscope (LSM880; ZEISS, Oberkochen, Germany) at the Instrument Analysis Center of Shenzhen University.

### 2.11 Xenograft mouse model

The animal experiments of constructing the xenograft mouse model were approved by the Animal Research Ethics Committee of Shenzhen University (license number: AEWC201412003). Male C57BL/6J mice (8 weeks old) were purchased from Guangzhou Yancheng Biotechnology Co. (Guangzhou, China), Ltd. C57BL/6J mice were randomly divided into two groups. *Siah1α* knockdown MC38 cells and the parental counterparts were injected subcutaneously into the right back of the mice (1 × 10^6^ cells per mouse, 10 mice per group), the growth of tumor was observed and the size of tumor was measured every 3 days. When the tumor volume reached 50 mm^3^, each group of mice was randomly divided into two groups and injected intraperitoneally with 10 mg·kg^−1^ SR3029 or vehicle (10% DMSO/40% PEG‐300/5% Tween‐20/45% saline) every 3 days respectively. Tumor volume was calculated by the formula: 0.5 × length × width^2^. After 9 days of drug treatment, mice were sacrificed, tumors were collected, weighed, and photographed. These mice were housed under standard laboratory conditions and were acclimatized to conditions for 1 week.

### 2.12 Histology and immunohistochemistry analyses

The tumor tissues were fixed with 4% paraformaldehyde for 48 h, then embedded with paraffin and made into 4 μm thick sections before H&E staining and immunohistochemical staining. The primary antibodies used for immunohistochemical staining were as follows: anti‐AXIN1 (2087S) and anti‐CyclinD1 (2978S) were purchased from Cell Signaling Technology.

### 2.13 Statistical analyses

Statistical analyses were carried out with graphpad prism 8.3.0 (GraphPad Software, Bethesda, MD, USA). The data were analyzed by Student's *t* test or Two‐way ANOVA. Results are presented as mean ± standard deviation (SD). Statistical significance was set at *P* < 0.05.

## 3 Results

### 3.1 CK1δ/ε inhibitors stabilizes AXIN1 protein in CRC cells

**Fig. 1:**
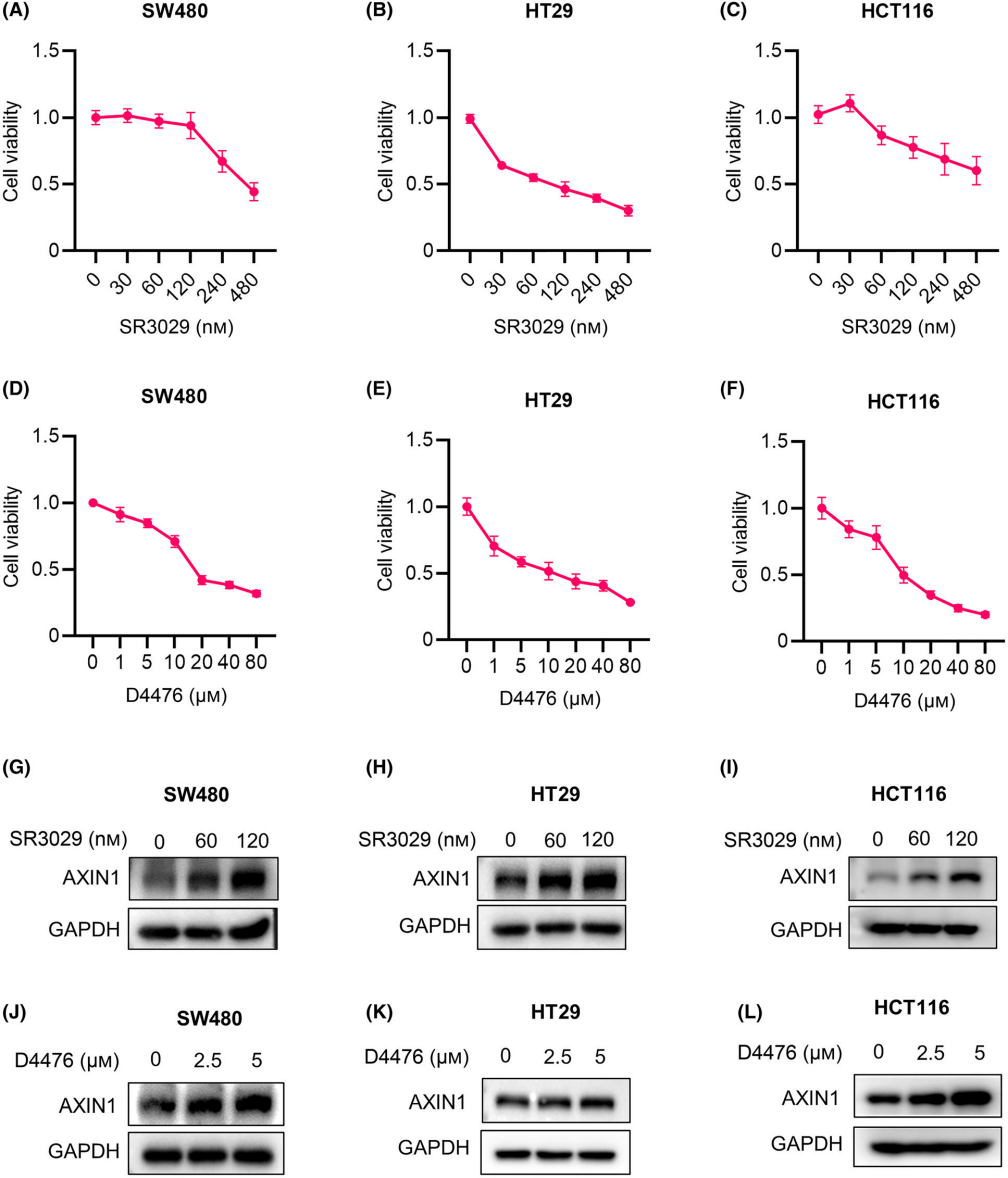
CK1δ/ε inhibitors induce AXIN1 protein stabilization in colorectal cancer cells. (A–C) The MTT assay was performed to assess the cell viability of SW480, HT29 and HCT116 cells exposed to different concentrations of SR3029 (0, 30, 60, 120, 240 and 480 nm) for 24 h (*n* = 5 independent experiments). (D–F) The MTT assay was performed to assess the viability of SW480, HT29 and HCT116 cells exposed to different concentrations of D4476 (0, 1, 5,10, 20, 40 and 80 μm) for 24 h (*n* = 5 independent experiments). (G–I) Immunoblot analysis of lysates of SW480, HT29 and HCT116 cells treated with 0, 60 and 120 nm SR3029 for 24 h before harvesting. (J–L) Immunoblot analysis of lysates of SW480, HT29 and HCT116 cells treated with 0, 2.5 and 5 μm D4476 for 24 h before harvesting. Shown is one representative of three independent experiments. Values are shown as means ± SD.

We first examined the impacts of CK1δ/ε inhibitors on the viability of CRC cells. The CRC SW480, HT29 and HCT116 cells were treated with different concentrations of the CK1δ/ε‐specific inhibitors SR3029 and D4476. Both inhibitors suppressed the viability of SW480, HT29, and HCT116 cells in a concentration‐dependent manner (Fig. 1A–F). We next determined the effect of CK1δ/ε inhibitors on the expression of some crucial components of Wnt signaling in CRC cells. As shown in Fig. S1A, treatment with SR3029 had little effect on the expression of LRP6 and GSK3β (Fig. S1A). Interestingly, we observed that this inhibitor markedly enhanced the protein level of AXIN1 in SW480 cells (Fig. S1A), suggesting that the CK1δ/ε inhibitors may suppress the viability of CRC cells through modulating the AXIN1 expression. In order to confirm these results, we checked whether other CK1δ/ε inhibitors have also any effect on the AXIN1 expression in CRC cells. The results showed that treatment with SR3029, D4476 and PF670462 (another CK1δ/ε‐specific inhibitor) significantly increased the protein level of AXIN1 without effect on its mRNA levels in SW480, HT29, and HCT116 cells (Fig. 1G–L and Fig. S1A–G). These results suggest that CK1δ/ε might be participate in the regulation of AXIN1 stability in CRC cells.

To determine the role of AXIN1 in CK1δ/ε‐mediated growth of CRC cells, we knocked down the AXIN1 expression in SW480, HT29, and HCT116 cells using lentivirus‐mediated shRNAs. In all three cell lines, treatment with either SR3029 or D4476 had less effect on cell viability in the cells with AXIN1 knockdown compared with parental cells (Fig. S2A–I), suggesting that AXIN1 might be an important player for CK1δ/ε‐mediated growth of CRC cells.

### 3.2 CK1ε regulates the protein expression of AXIN1

**Fig. 2:**
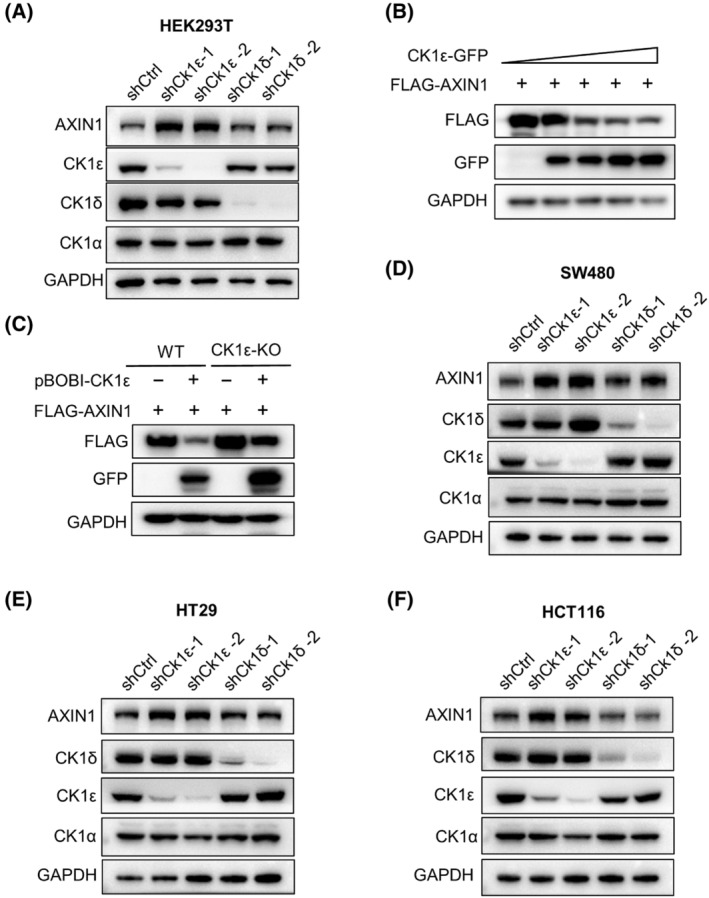
CK1ε regulates the protein expression of AXIN1. (A) Immunoblot analysis of lysates of HEK293T cells infected with shCtrl, shCK1ε‐1, shCK1ε‐2, shCK1δ‐1 and shCK1δ‐2 lentivirus. (B) Immunoblot analysis of lysates of HEK293T cells transfected with FLAG‐AXIN1 and gradient concentrations of GFP‐CK1ε (0, 0.1, 0.25, 0.5 and 1 μg) plasmid. (C) Immunoblot analysis of lysates of control cells and CK1ε‐deficient HEK293T cells (infected with lentivirus containing pBOBI‐CK1ε or pBOBI vector as a control) transfected with indicated plasmids encoding Flag‐tagged AXIN1. (D–F) Immunoblot analysis of lysates of SW480, HT29 and HCT116 cells infected with shCtrl, shCK1ε‐1, shCK1ε‐2, shCK1δ‐1 and shCK1δ‐2 lentivirus. Shown is one representative of three independent experiments.

CK1ε and CK1δ are the most closely related, sharing the highest degree of sequence similarity in the CK1 family. We next investigated the effects of CK1ε and CK1δ on AXIN1 expression. Lentivirus‐mediated shRNAs were used to silence the expression of CK1ε or CK1δ in HEK293T cells. As shown in Fig. 2A, knockdown of *CSNK1E* elevated the protein level of AXIN1, whereas knockdown of *CSNK1D* had little effect (Fig. 2A). Consistently, overexpression of CK1ε decreased the protein level of AXIN1 in a dose‐dependent manner in HEK293T cells transfected with Flag‐AXIN1 expression vector along with gradient concentrations of CK1ε‐GFP expression plasmid (Fig. 2B). Moreover, deficiency of *CSNK1E* in HEK293T cells increased exogenous AXIN1 level, while this effect was rescued by re‐expression of CK1ε (Fig. 2C). We also examined the effect of CK1ε on AXIN1 expression in CRC cells. Knockdown of *CSNK1E* but not knockdown of *CSNK1D* greatly increased the protein stability of AXIN1, but had little effect on the mRNA level of AXIN1 in SW480, HT29 and HCT116 cells (Fig. 2D–F and Fig. S3A–C). These results demonstrated that CK1ε was implicated in the regulation of AXIN1 stability.

### 3.3 Knockdown of *CSNK1E* attenuates the Wnt/β‐catenin signaling pathway and inhibits the viability and colon formation in CRC cells

**Fig. 3:**
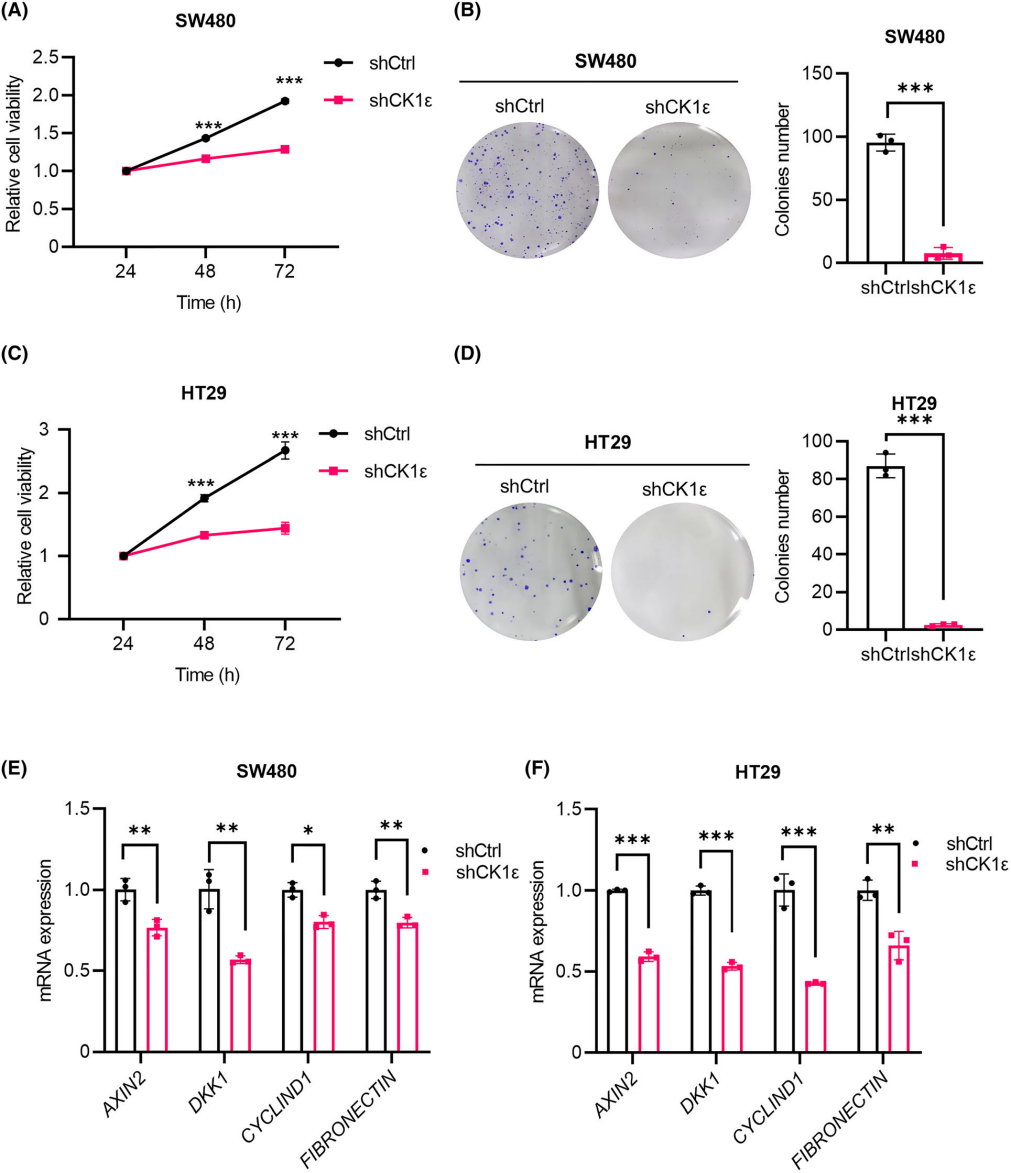
*CSNK1E* knockdown reduces the viability and colon formation ability in CRC cells through attenuating Wnt/β‐catenin signaling. SW480 and HT29 cells were infected with the shCtrl or shCK1ε lentivirus respectively. (A, C) MTT assay was used to detect cell viability (*n* = 5 independent experiments). (B, D) Colony formation assay was performed to evaluate colony formation ability. Graphical representation of quantitative data showed the relative number of colonies formed (*n* = 3 independent experiments). (E, F) RT‐qPCR was performed to detect the mRNA expression of Wnt target genes (*CYCLIND1*, *AXIN2*, *DKK1*, and *FIBRONECTIN*). Quantification of mRNA level was normalized to *GAPDH* (*n* = 3 independent experiments). Values are shown as means ± SD. *P < 0.05, **P < 0.01, ***P < 0.001; Student's *t* test.

We next assessed the effect of CK1ε on the biological behaviors of CRC cells. Silencing CK1ε using lentivirus‐mediated shRNAs markedly decreased the viability of SW480 and HT29 cells (Fig. 3A,C). Using a colony formation assay, we showed that depletion of *CSNK1E* reduced the capability of colony formation in SW480 and HT29 cells (Fig. 3B,D). Furthermore, knockdown of *CSNK1E* downregulated the expression of Wnt target genes *AXIN2*, *DKK1*, *CYCLIND1*, and *FIBRONECTIN* (Fig. 3E,F). Treatment with SR3029 also downregulated the expression of *AXIN2* (Fig. S4A,B). These results suggest that CK1ε may play an important role in CRC cell viability and colony formation ability. Concerning that silencing CK1ε resulted in a decrease in *AXIN2* mRNA level in CRC cells through downregulating Wnt/β‐catenin signaling, we focus on CK1ε function in regulation of AXIN1 protein stability in subsequent study.

### 3.4 CK1ε regulates AXIN1 stability via the ubiquitin‐proteasome pathway

**Fig. 4:**
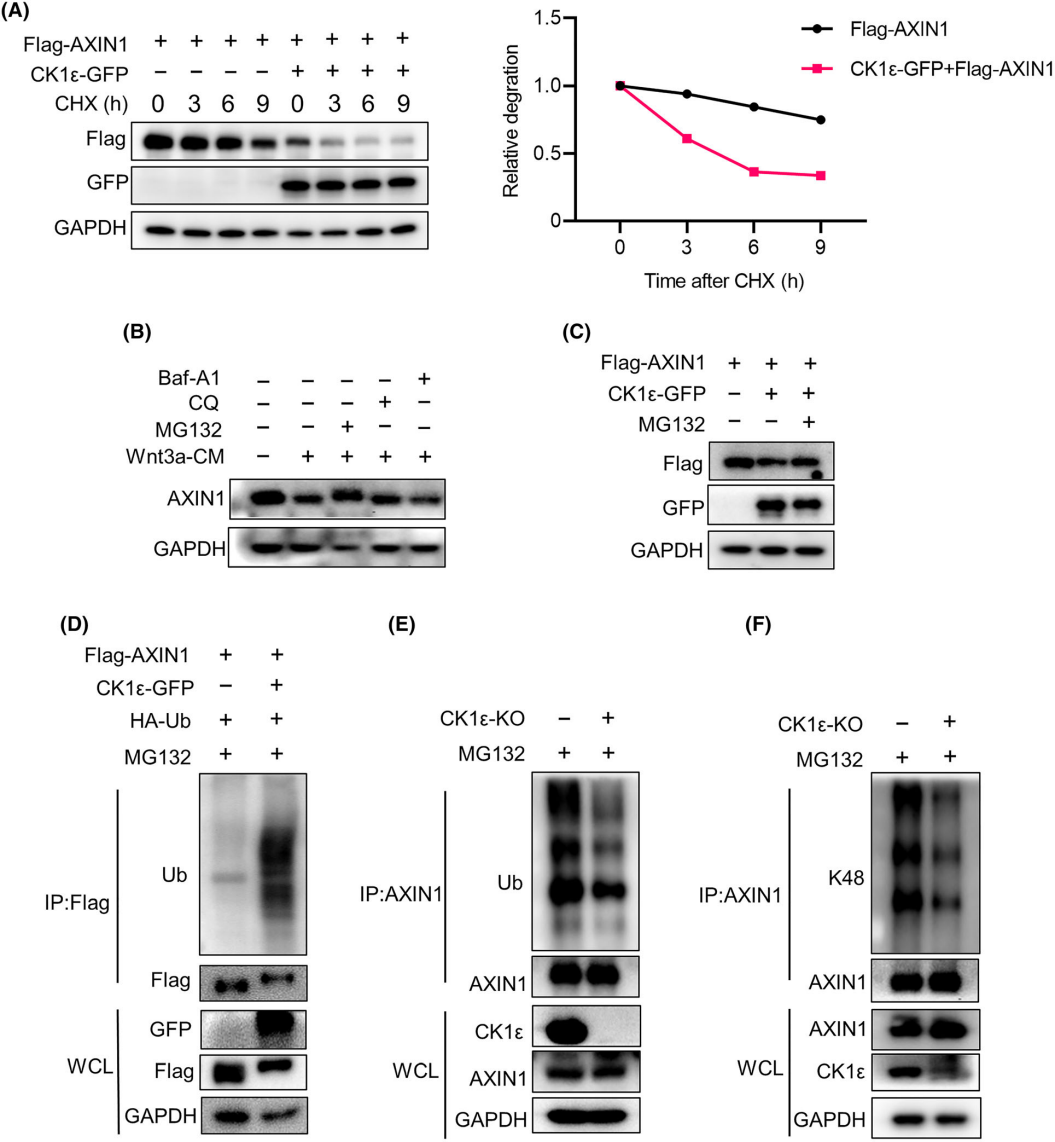
CK1ε regulates AXIN1 stability via the ubiquitin‐proteasome pathway. (A) Immunoblot analysis of lysates of HEK293T cells transfected with various indicated plasmids encoding FLAG‐AXIN1 and GFP‐CK1ε and then treated with 100 mg·mL^−1^ CHX for 0, 3, 6, and 9 h before harvesting. (B) Immunoblot analysis of lysates of HEK293T cells treated with or without 10 μm MG132 for 8 h, 50 nm CQ, 10 nm Baf‐A1 for 24 h with Wnt3a conditioned medium (Wnt3a‐CM) before harvesting. (C) Immunoblot analysis of lysates of HEK293T cells transfected with various indicated plasmids encoding FLAG‐AXIN1 and GFP‐CK1ε and then treated with 10 μm MG132 or DMSO for 8 h before harvesting. (D) Immunoprecipitation and immunoblot analysis of lysates of HEK293T cells transfected with various indicated plasmids encoding FLAG‐AXIN1, GFP‐CK1ε and HA‐Ub, and then treated with 10 μm MG132 for 8 h before harvesting. (E, F) Immunoprecipitation and immunoblot analysis of lysates of control or *CSNK1E*‐deficient cells treated with 10 μm MG132 for 8 h before harvesting. Shown is one representative of three independent experiments.

To evaluate the regulation of AXIN1 stability by CK1ε, HEK293T cells treated with the protein synthesis inhibitor cycloheximide (CHX). CHX treatment decreased the protein level of AXIN1, and overexpression of CK1ε further reduced the half‐life of AXIN1 protein (Fig. 4A). As expected, treatment with Wnt3a conditioned medium (Wnt3a‐CM) reduced endogenous AXIN1 level, while this process was rescued by proteasome inhibitor MG132 but not by lysosomal inhibitors CQ and Baf‐A1, suggesting that Wnt‐induced AXIN1 degradation was mediated by the proteasome (Fig. 4B). Similarly, CK1ε‐regulated degradation of exogenous AXIN1 was also rescued by MG132 (Fig. 4C). Moreover, overexpression of CK1ε enhanced the ubiquitination of AXIN1 (Fig. 4D). In contrast, *CSNK1E* deficiency or treatment with CK1δ/ε inhibitor SR3029 markedly diminished the AXIN1 ubiquitination in CRC cells (Fig. 4E and Fig. S5A,B). To explore the ubiquitin chain formation linked to AXIN1, we used a specific ubiquitin K48 antibody to detect the ubiquitination of AXIN1, and demonstrated that knockout of *CSNK1E* significantly weakened the K48‐linked ubiquitination of AXIN1 (Fig. 4F). These results indicate that CK1ε promoted the degradation of AXIN1 via the K48‐mediated ubiquitin‐proteasome pathway.

### 3.5 CK1ε promotes the interaction of SIAH1 with AXIN1

**Fig. 5:**
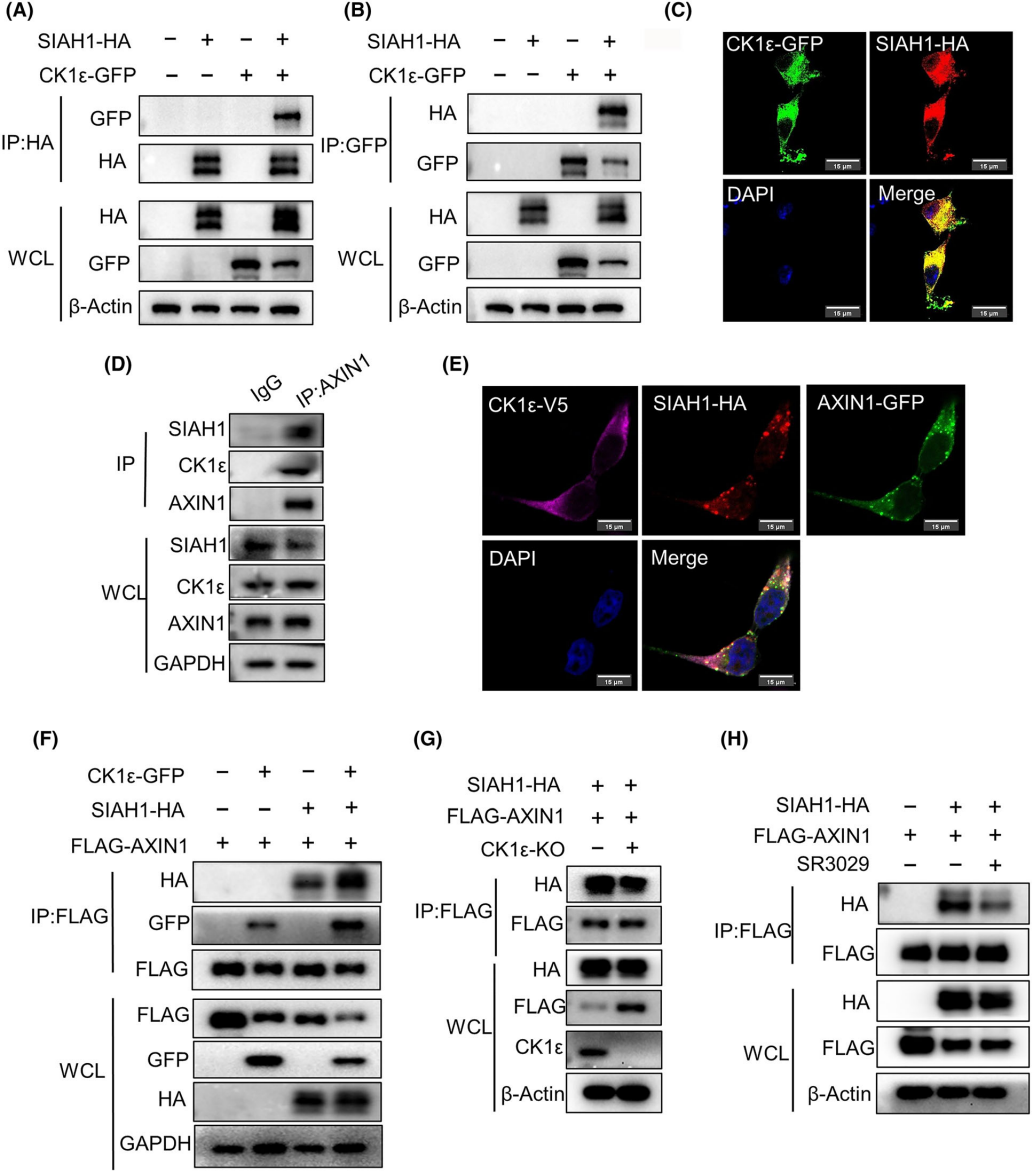
CK1ε promotes the interaction of SIAH1 with AXIN1. (A, B) Immunoprecipitation and immunoblot analyses of lysates of HEK293T cells transfected with various indicated plasmids encoding GFP‐CK1ε and HA‐SIAH1. (A) IP was performed with FLAG‐tagged beads. (B) IP was performed with GFP‐tagged beads. (C) Confocal microscopy of HEK293T cells transfected with plasmids encoding GFP‐CK1ε and HA‐SIAH1, scale bars = 15 μm. (D) Immunoprecipitation and immunoblot analyses of lysates of HEK293T cell were performed using control IgG or anti‐AXIN1 antibody. (E) Confocal microscopy of HEK293T cells transfected with plasmids encoding V5‐CK1ε, HA‐SIAH1 and GFP‐AXIN1, scale bars = 15 μm. (F) Immunoprecipitation and immunoblot analyses of lysates of HEK293T cells transfected with various indicated plasmids encoding GFP‐CK1ε, HA‐SIAH1 and FLAG‐AXIN1. (G) Immunoprecipitation and immunoblot analyses of lysates of *CSNK1E*‐deficient HEK293T cells or control cells transfected with indicated plasmids encoding HA‐SIAH1 and FLAG‐AXIN1. (H) Immunoprecipitation and immunoblot analyses of lysates of HEK293T cells transfected with various indicated plasmids encoding HA‐SIAH1 and FLAG‐AXIN1 and treated with DMSO or SR3029 (100 nm) 24 h. Shown is one representative of three independent experiments.

SIAH has been shown to facilitate Wnt‐induced degradation of AXIN1 by interacting with the VxP motif of AXIN [23]. We thus checked whether the association of CK1ε with SIAH1 occurred in the process of Wnt‐induced AXIN1 degradation. A co‐immunoprecipitation assay was performed using HEK293T cells transfected with GFP‐CK1ε and HA‐SIAH1. Our results showed that SIAH1 could interact with CK1ε (Fig. 5A,B). We also observed that CK1ε was co‐localized with SIAH1 following their co‐transfection in HEK293T cells (Fig. 5C). Moreover, endogenous interaction of CK1ε, SIAH1 and AXIN1 was detected by co‐immunoprecipitation in HEK293T cells (Fig. 5D). Additionally, partial colocalization of CK1ε‐V5, SIAH1‐HA and AXIN1‐GFP was observed in co‐transfected HEK293T cells (Fig. 5E). These results clearly showed that CK1ε, SIAH1 and AXIN1 coexisted in the same protein complex. Importantly, we noted that co‐expression of CK1ε markedly promoted the binding of SIAH1 to AXIN1 in HEK293T cells (Fig. 5F). Consistent with this result, deficiency of *CSNK1E* or treatment with CK1δ/ε inhibitor SR3029 resulted in decreased binding of SIAH1 to AXIN1 (Fig. 5G,H). Furthermore, we examined whether CK1ε has any effect on AXIN1 phosphorylation. HEK293T cells were transfected with AXIN1 expression vector with or without an expression plasmid for CK1ε. Western blot analysis showed that overexpression of CK1ε markedly elevated the level of phosphorylated AXIN1 (Fig. S6A), detected by a pan phospho‐serine antibody. In addition, the interaction between AXIN1 and SIAH1 was diminished upon treatment with λ‐phosphatase (λ‐PPase) (Fig. S6B). Together, these results suggest that CK1ε could interact with SIAH1 and AXIN1, and promote the interaction between SIAH1 and AXIN1 through AXIN1 phosphorylation. However, it remains unclear whether this interaction is direct or indirect.

### 3.6 CK1ε synergizes with SIAH1 to promote AXIN1 ubiquitination and degradation

**Fig. 6:**
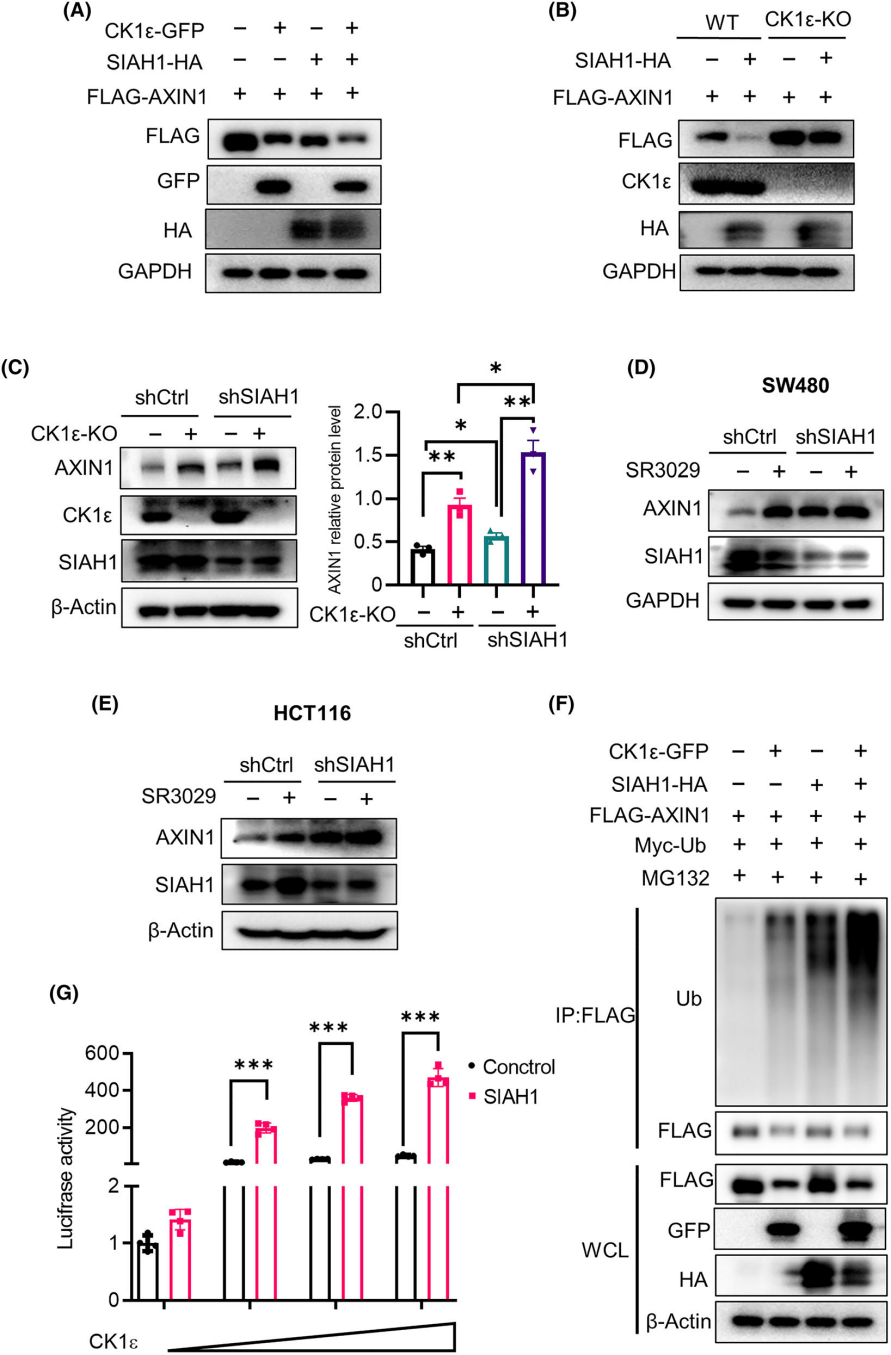
CK1ε synergizes with SIAH1 to promote AXIN1 ubiquitination and degradation. (A) Immunoblot analysis of lysates of HEK293T cells transfected with various indicated plasmids encoding HA‐SIAH1, GFP‐CK1ε and FLAG‐AXIN1. (B) Immunoblot analysis of lysates of *CSNK1E*‐deficient HEK293T cells or control cells transfected with indicated plasmids encoding HA‐SIAH1 and FLAG‐AXIN1. (C) Immunoblot analysis of lysates of *CSNK1E*‐deficient HEK293T cells or control cells infected with shCtrl or shSIAH1 lentivirus (*n* = 3 independent experiments). (D, E) Immunoblot analysis of lysates of SW480 and HCT116 cells infected with shCtrl or shSIAH1 lentivirus and then treated with 100 nm SR3029 for 24 h before harvesting. (F) Immunoprecipitation and immunoblot analyses of lysates of HEK293T cells transfected with various indicated plasmids encoding HA‐SIAH1, FLAG‐AXIN1 and GFP‐CK1ε and then treated with 10 μm MG132 for 8 h before harvesting. (G) The SuperTOPFlash reporter gene was transfected into HEK293T cells with SIAH1 or control plasmids together with gradient concentrations of CK1ε plasmid (*n* = 4 independent experiments). Shown is one representative of at least three independent experiments. Values are shown as means ± SD. *P < 0.05, **P < 0.01, ***P < 0.001; Student's *t* test.

We further evaluate the effect of CK1ε on SIAH1‐mediated AXIN1 degradation. Overexpression of CK1ε markedly enhanced the degradation of AXIN1 by SIAH1 in transfected HEK293T cells (Fig. 6A), while depletion of *CSNK1E* abolished the AXIN1 degradation by SIAH1 (Fig. 6B). Silencing *SIAH1* in *CSNK1E*‐deificient (CK1ε‐KO) cells caused increased protein level of AXIN1 compared to wild‐type (WT) control cells (Fig. 6C and Fig. S7A). Moreover, knockdown of *SIAH1* in HCT116 and SW480 cells increased the protein level of AXIN1 and synergized with SR3029 to enhance AXIN1 expression at the protein level (Fig. 6D,E). Additionally, co‐expression of CK1ε with SIAH1 and AXIN1 significantly promoted AXIN1 polyubiquitination in HEK293T cells (Fig. 6F). We also tested the effect of SIAH1 and/or CK1ε on Wnt/β‐catenin signaling. As illustrated in Fig. 6G, the expression of SIAH1 and CK1ε synergistically enhanced the luciferase activity of the SuperTOPFlash reporter (Fig. 6G). These results indicate that CK1ε could synergize with SIAH1 to promote AXIN1 ubiquitination and degradation, which positively regulate the Wnt/β‐catenin signaling pathway.

AXIN1 V383A mutation in the SIAH‐binding motif has been reported to abolish its SIAH‐binding capability [23]. We constructed AXIN1 point mutant V383A. Our results showed that Axin1 V383A mutation partially abrogated CK1ε‐mediated degradation of AXIN1 (Fig. S7B), suggesting that the SIAH‐binding motif of AXIN1 may play an important role in CK1ε/SIAH‐mediated stability of AXIN1.

Since tankyrase could regulate the stability of AXIN by mediating poly‐ADP‐ribosylation of AXIN, we examined the effect of tankyrase inhibitor XAV939 on CK1ε‐mediated degradation of AXIN1. Treatment with XAV939 obviously enhanced the level of AXIN1. CK1ε overexpression strongly reduced AXIN1 levels in the absence or presence of 10 μm XAV939 (Fig. S7C). In addition, we constructed a deletion mutant of AXIN1 that lacks the N‐terminal 20–29 amino acids (Δ20–29), resulting in a reduced binding capability to tankyrase. This mutation had little effect on CK1ε‐mediated degradation of AXIN1 (Fig. S7D). Collectively, these results demonstrated that CK1ε was implicated in the regulation of AXIN1 stability in a tankyrase‐independent manner.

### 3.7 CK1ε cooperates with SIAH1 to promote the viability and colony formation ability of CRC cells through regulating Wnt/β‐catenin signaling

**Fig. 7:**
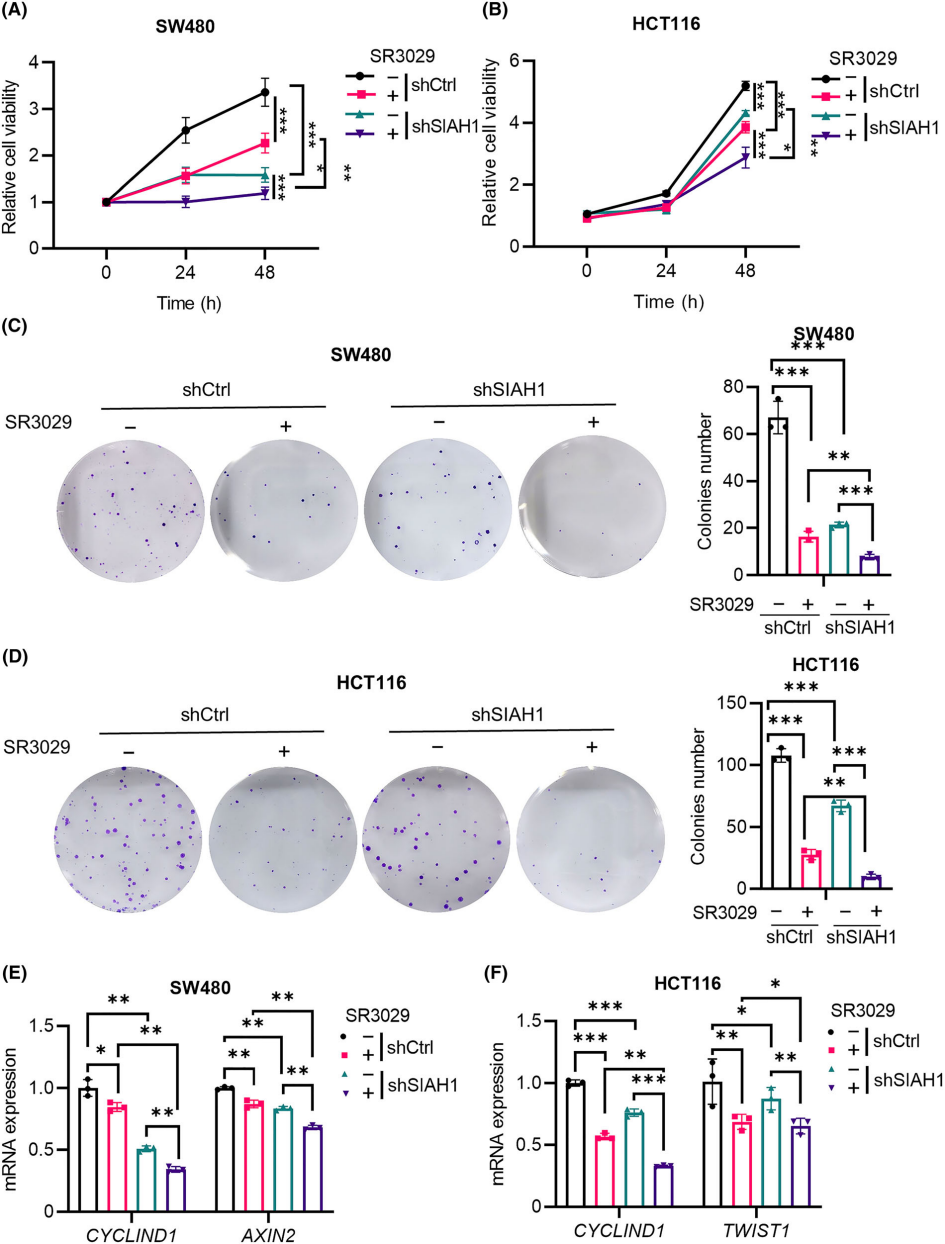
SR3029 collaborates with knockdown of *SIAH1* to inhibit cell viability and colony formation ability via downregulating Wnt/β‐catenin signaling in CRC cells. SW480 and HCT116 cells were infected with the shCtrl or shSIAH1 lentivirus, then treated with DMSO or SR3029 (100 nm) for 24 h (A, B, E, F), 48 h (A, B) or 12 days (C, D), respectively. (A, B) MTT assay was used to detect cell viability (*n* = 5 independent experiments). (C, D) Colony formation assay was performed to evaluate the cell proliferation. Graphical representation of quantitative data showed the relative number of colonies formed (*n* = 3 independent experiments). (E, F) RT‐qPCR was performed to detect mRNA expression of Wnt target genes (*CYCLIND1*, *AXIN2*, *TWIST*). Quantification of mRNA level was normalized to *GAPDH* (*n* = 3 independent experiments). Values are shown as means ± SD. *P < 0.05, **P < 0.01, ***P < 0.001; Student's *t* test.

We next investigated the effects of CK1ε and SIAH1 on the viability of CRC cells. The human CRC SW480 and HCT116 cells were used to investigate the effects of *SIAH1* knockdown and the CK1δ/ε inhibitor SR3029 on the biological characteristics of CRC cells. Either *SIAH1* knockdown or SR3029 treatment displayed an obviously decrease in the viability and the capability of colony formation in SW480 and HCT116 cells (Fig. 7A–D). Knockdown of *SIAH1* combined with SR3029 treatment was more effective in reducing cell viability and colony formation ability in both CRC cell lines (Fig. 7A–D). Moreover, decreased mRNA expression of Wnt target genes (*CYCLIND1*, *AXIN2*, and *TWIST*) was detected by real‐time PCR in CRC cells with *SIAH1* knockdown and/or SR3029 treatment (Fig. 7E,F). These results suggest that pharmacological inhibition of CK1ε could collaborate with SIAH1 knockdown to inhibit the viability and colony formation ability of CRC cells via regulating the Wnt/β‐catenin pathway.

In addition, overexpression of CK1ε or SIAH1 increased cell viability, and simultaneous expression of CK1ε and SIAH1 further promoted the viability of SW480 and HT29 cells (Fig. S8A,C). Meanwhile, overexpression of CK1ε or SIAH1 upregulated the mRNA expression of Wnt target gene *CYCLIND1* in SW480 and HT29 cells. Simultaneous expression of CK1ε and SIAH1 further enhanced mRNA expression of *CYCLIND1* compared with CRC cells transfected with CK1ε or SIAH1. (Fig. S8B,D). These results indicate that simultaneous expression of CK1ε and SIAH1 enhanced the viability of CRC cells.

### 3.8 SR3029 enhances the suppressive effect of *Siah1α* knockdown on colorectal tumor growth

**Fig. 8:**
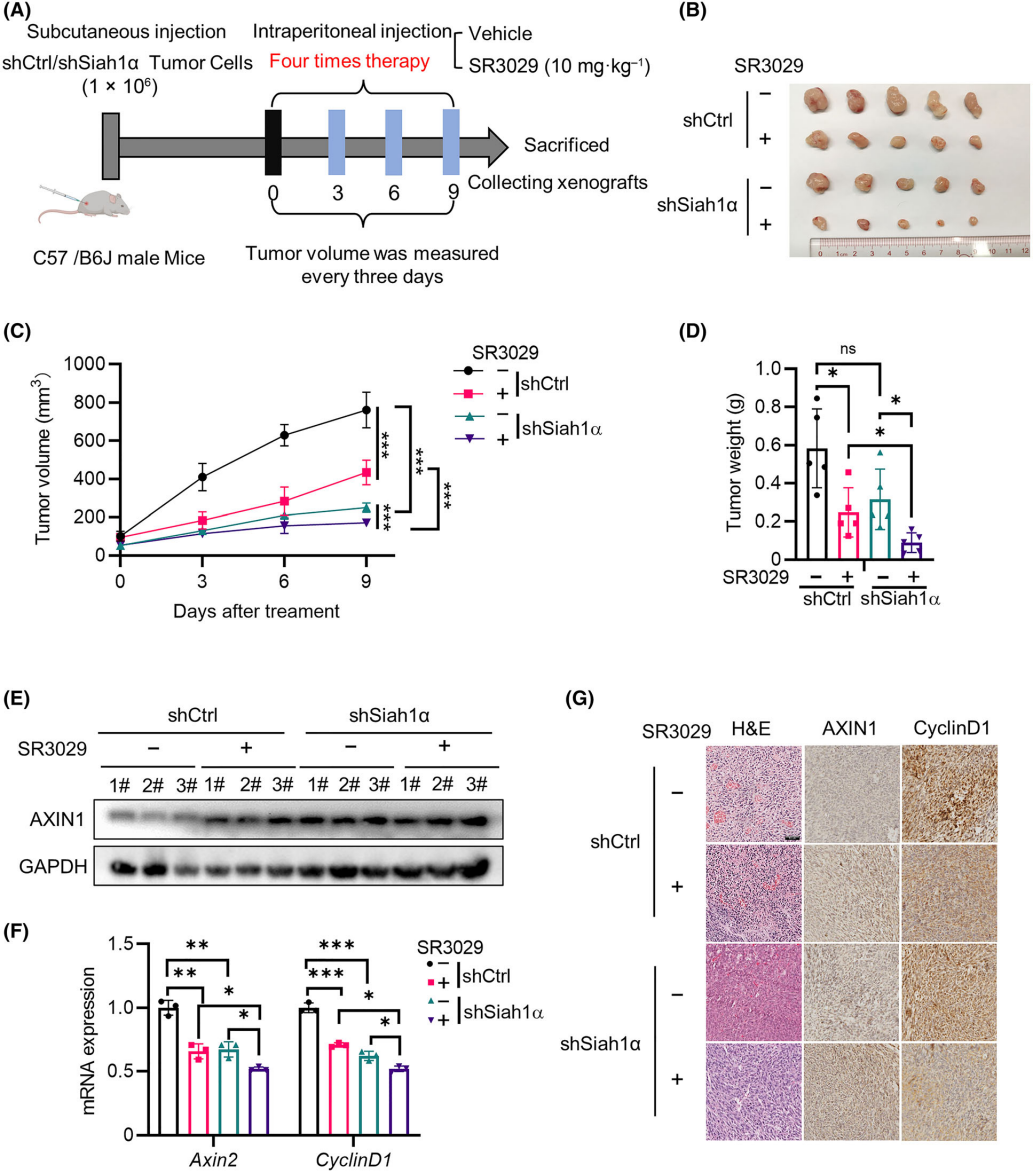
SR3029 enhances the suppressive effect of *Siah1α* knockdown on colorectal tumor growth *in vivo* by stabilizing AXIN1. (A) CRC xenograft model. *Siah1α*‐deficient MC38 cells and parental counterparts were subcutaneously (s.c.) implanted into the right back of 8‐week‐old male C57BL/6J mice to generate a xenograft tumor model. When the tumors reached about 50 mm^3^, mice were randomly divided into two groups and intraperitoneally (i.p.) injected with the vehicle (10% DMSO/40% PEG‐300/5% Tween‐80/45% saline) or SR3029 (10 mg·kg^−1^) every 3 days. Tumor sizes were measured with a caliper and tumor volumes were calculated using the formula: 0.5 × length × width^2^. At 9 days after SR3029 treatment, mice were sacrificed, and tumors were collected and photographed. (B) Images of tumors from each experimental group. (C) Mean tumor volume in each experimental group (*n* = 5 independent experiments). (D) Mean tumor weight in each experimental group (*n* = 5 independent experiments). (E) Immunoblot analysis of AXIN1 in each experimental group. (F) RT‐qPCR analysis of Wnt target genes (*CyclinD1*, *Axin2*) in each experimental group (*n* = 3 independent experiments). (G) H&E, AXIN1 and CYCLIND1 staining of each experimental group, scale bars = 50 μm. Shown is one representative of three independent experiments. Values are shown as means ± SEM. *P < 0.05, **P < 0.01, ***P < 0.001; Two‐way ANOVA for (C); Student's *t* test for (D) and (F).

To further explore the effects of CK1ε and SIAH1 on tumor growth *in vivo*, *Siah1α*‐knockdown MC38 cells and their parental counterparts were subcutaneously injected into C57BL/6J mice to generate a xenograft tumor model. As shown in Fig. S9A,B, the *Siah1α* mRNA and protein levels were knocked down in mouse MC38 cells (Fig. S9A,B). Either silencing *Siah1α* or SR3029 treatment decreased the protein level of β‐catenin (Fig. S9C). When the tumors reached about 50 mm^3^, mice were randomly divided into two groups and intraperitoneally injected with the vehicle or SR3029 (10 mg·kg^−1^) every 3 days (Fig. 8A). In the xenograft tumor model, reduced tumor growth in mice silencing *Siah1α* was observed (Fig. 8B–D). Mice with *Siah1α* knockdown and SR3029 administration had the slowest tumor growth, but had no effect on body weight (Fig. 8B–D and Fig. S9D,E), concomitant with increased protein level of AXIN1 and decreased expression of Wnt target genes (*CyclinD1*, *Axin2*) (Fig. 8E–G). Collectively, these results illustrated that *Siah1α* knockdown combined with SR3029 administration synergistically inhibits CRC growth through stabilizing AXIN1.

## 4 Discussion

The colorectal cancer (CRC) is the third most commonly diagnosed malignancies in the world, and the therapeutic effect, including surgical resection and chemoradiotherapy, remains relatively poor [45]. Therefore, it has great clinical significance to explore new therapeutic targets for treatment of CRC. Many studies have shown that the Wnt/β‐catenin signaling pathway is precisely regulated, and its abnormal activation plays an important role in tumorigenesis and progression of CRC [46, 47, 48]. *AXIN1* is a tumor suppressor and a crucial negative regulator of the Wnt/β‐catenin signaling pathway. Abnormal expression of AXIN1 protein has been shown to be associated with various tumors, including CRC [49, 50]. The protein level of AXIN1 is regulated by the poly‐ADP‐ribosylating enzymes tankyrase 1 and tankyrase 2. The tankyrases interact with AXIN1 and enhance its degradation through the ubiquitin‐proteasome pathway. The tankyrase inhibitor XAV939 could reduce the expression of β‐catenin by upregulating AXIN1, increase apoptosis induced by 5‐Fu and suppress tumor growth in CRC [51]. Mizutani et al. reported that TNKS inhibitors G007‐LK and RK‐287107 could potentiate proliferative inhibition of BET inhibitors through decreasing β‐catenin expression in colorectal cancer cells [52]. In this study, we found that CK1δ/ε inhibitors could enhance AXIN1 protein level via targeting CK1ε in CRC cells. We thus explored the role and mechanism of CK1δ and CK1ε in regulation of AXIN1 protein stability. Our results showed that CK1ε could promote the interaction of SIAH1 with AXIN1, resulting in AXIN1 degradation in the ubiquitin‐proteasome pathway. Genetic or pharmacological inhibition of CK1ε and knockdown of *SIAH1* downregulated the expression of Wnt‐dependent genes and restrained tumorigenesis and progression of CRC through inhibiting Wnt/β‐catenin signaling. This study revealed a novel mechanism by which the CK1ε‐SIAH1 axis regulates AXIN1 stability and tumorigenesis in CRC. AXIN1 is a key scaffolding protein of the β‐catenin destruction complex. The AXIN1 protein stability was sensitive and regulated by the Wnt3a stimulation or other components of Wnt/β‐catenin signaling [12, 53], suggesting that AXIN1 stability may have a significant impact on Wnt/β‐catenin signaling. In our results, we found that the decreased expression of AXIN1 induced by Wnt3a‐CM was rescued by proteasome inhibitor MG132 but not lysosomal inhibitors CQ and Baf‐A1, suggesting that Wnt/β‐catenin signaling‐induced AXIN1 degradation was depended on the ubiquitin‐proteasome pathway. Although it has been reported that several E3 ubiquitin ligase (such as SIAH1 and RNF146) could promote AXIN1 ubiquitination and degradation [18, 23, 54]. However, there still lack a clear understanding of the molecular mechanism of regulating the stability of AXIN1 protein, and this process need further investigation.

AXIN2 is a homolog of AXIN1 and a well‐known target gene of the Wnt/β‐catenin pathway. Although CK1ε may mediate the stability of AXIN1 and AXIN2 via a similar mechanism, our real‐time PCR results demonstrated that silencing CK1ε or treatment with CK1δ/ε inhibitors downregulated the mRNA expression of *AXIN2* through inhibiting Wnt/β‐catenin signaling in CRC cells. Reduced AXIN2 mRNA levels will lead to less AXIN2 protein that can potentially be stabilized through our proposed mechanism, making it more complicated to determine the contribution of AXIN2.

CK1ε, one of the casein kinase 1 family members, is a conserved serine/threonine‐specific protein kinase [55, 56]. Casein kinase 1 family members have been involved in carcinogenic process by regulating Wnt/β‐catenin and Hedgehog signaling pathways [55]. When Wnt/β‐catenin signaling was activated, CK1δ/ε could directly interact and phosphorylate DVL, facilitate the accumulation and nuclear translocation of β‐catenin, resulting in promoting the expression of Wnt‐dependent genes [53, 57]. Ye et al. found that the expression of CK1ε protein was significantly higher in CRC tissues than that in adjacent noncancerous tissues. Depletion of CK1ε inhibited cell proliferation, and invasion of CRC cells through inhibition of Wnt/β‐catenin signaling [58]. CK1δ/ε inhibitor IC261 has been found to inhibit the growth of CRC cells and elevate the level of aerobic glycolysis [59]. Our previous study showed that CK1δ/ε could enhance the SKP2‐mediated ubiquitination and degradation of amino‐terminal enhancer of split (AES). CK1δ/ε inhibitor SR3029 treatment suppressed tumor growth via stabilizing AES in APC^min/+^ colorectal tumor organoids and patient‐derived colorectal tumor xenografts [60]. Here, we demonstrated that CK1ε exerted its oncogenic role in CRC occurrence and progression by regulating the stability of AXIN1. CK1ε promoted AXIN1 degradation by the ubiquitin‐proteasome pathway.

The E3 ubiquitin ligase SIAH1 targets diverse substrates for ubiquitin‐dependent degradation, which are implicated in many biological processes, such as proliferation, migration and invasion. Liu et al. [61] reported that SIAH1 could promote Akt phosphorylation and enhance the proliferation of non‐small cell lung cancer (NSCLC) via ubiquitinating and stabilizing Notch1 by proteasome pathway. SIAH1 has been found to promote AXIN1 ubiquitination and degradation and positively regulate Wnt signaling [23]. In this study, we demonstrated that CK1ε could potentiate the interaction between SIAH1 and AXIN1 and accelerate the SIAH1‐mediated AXIN1 ubiquitination and degradation. Our results suggest that CK1ε‐SIAH1‐AXIN1 signaling axis may be implicated in tumorigenesis of CRC. Actually, the interaction between CK1ε and AXIN1 has been reported by several previous studies [36, 62]. The carboxyl terminal sequence and the MEKK1‐interacting domain (MID) domain of AXIN1, as well as the carboxyl terminal domain of CK1ε were required for the interaction of CK1ε with AXIN1. However, how CK1ε potentiates the association of SIAH1 with AXIN1 remains to be further studied.

The MC38 cell line is a commonly used murine model for colorectal cancer. Previous studies showed that the β‐catenin‐dependent signaling is relatively low in MC38 cells [63]. We thus examined the effect of silencing *Siah1α* or CK1δ/ε inhibitor SR3029 on the protein levels of β‐catenin in MC38 cells. Our results showed that either silencing *Siah1α* or SR3029 treatment reduced the protein level of β‐catenin. In MC38 tumor‐bearing mice, *Siah1α* knockdown combined with SR3029 administration synergistically inhibits CRC growth, concomitant with increased protein level of AXIN1, and downregulation of Wnt target genes. These results indicated that the CK1ε/SIAH1‐mediated stability of AXIN1 may suppress tumor growth in a β‐catenin‐dependent fashion.

**Fig. 9:**
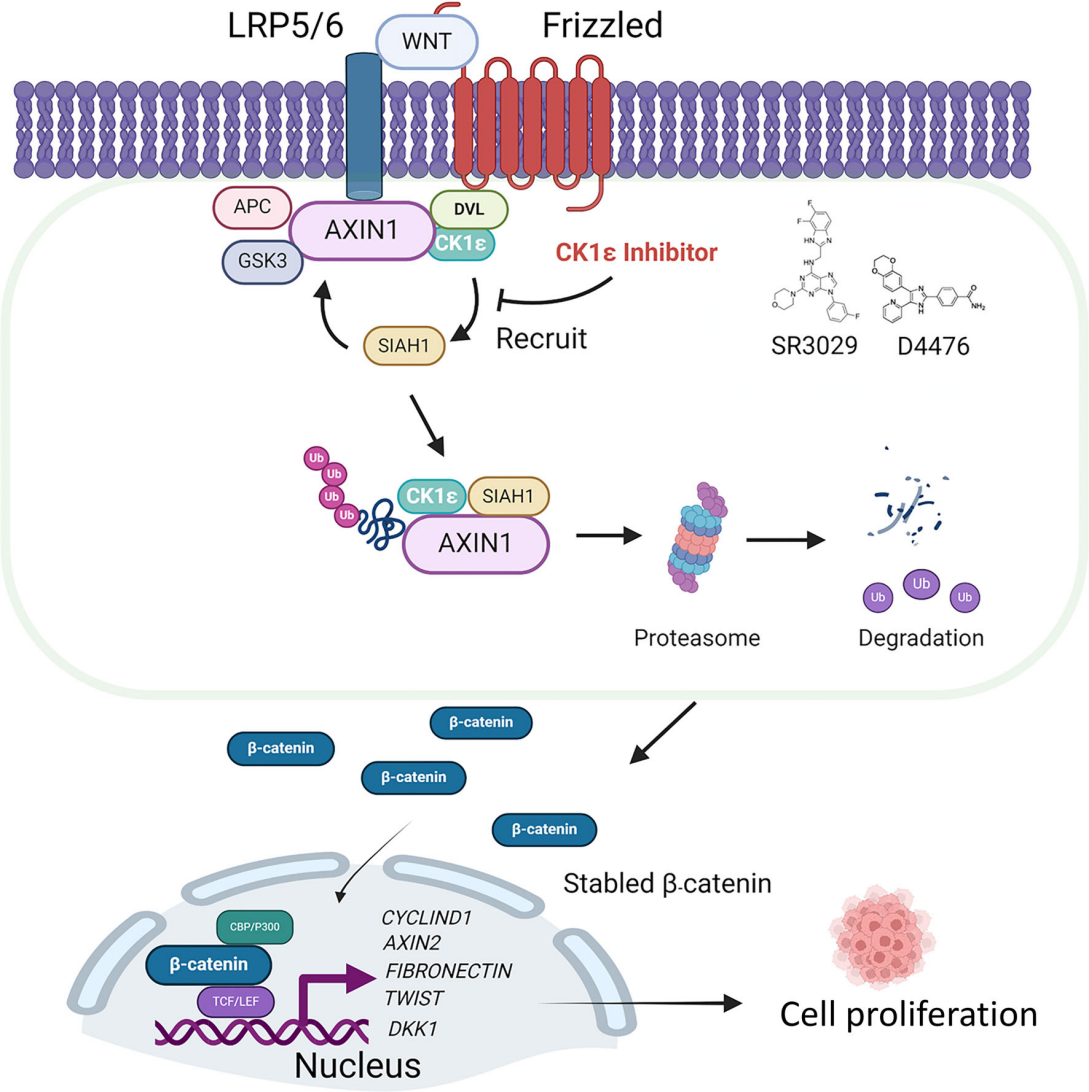
Molecular model depicting the CK1ε‐SIAH1‐AXIN1 regulatory module in CRC cells. Schematic representation of the role of CK1ε in the regulation of AXIN1 degradation via the ubiquitin‐proteasome pathway. CK1ε promoted AXIN1 degradation by the ubiquitin‐proteasome pathway via promoting the interaction of E3 ubiquitin ligase SIAH1 with AXIN1. CK1δ/ε inhibitors could significantly enhance AXIN1 protein level through targeting CK1ε.

As a kinase, CK1ε has significant advantages in the development of small molecule drugs. Our data showed that CK1δ/ε inhibitor SR3029 and D4476 obviously increased AXIN1 protein level and decreased the expression of Wnt target genes. What's more, treatment SR3029 suppressed tumor growth by stabilizing AXIN1 in xenograft tumor model. The present study identified a CK1ε‐SIAH1‐AXIN1 signaling axis for the regulation of AXIN1 protein stability, and this axis may play an important role in the tumorigenesis and progression of CRC (Fig. 9). Therefore, development of drugs targeting CK1ε and blocking this axis may be a feasible strategy for the treatment of CRC.

## 5 Conclusion

In this study, we found that the CK1δ/ε inhibitors could stabilize the AXIN1 protein through targeting CK1ε. We further showed that CK1ε regulated the protein level of AXIN1 by enhancing the binding of SIAH1 to AXIN1 and promoting K48‐linked ubiquitination‐mediated proteasome degradation of AXIN1. Knockdown of CK1ε negatively regulated the activation of Wnt/β‐catenin signaling and suppressed the proliferation of CRC cells. Overexpression of CK1ε and SIAH1 exerted synergistic effects on promoting the viability of CRC cells. In the mouse xenografts model, treatment with CK1δ/ε inhibitor SR3029 and knockdown of SIAH1 resulted in attenuated colorectal tumor growth via stabilizing AXIN1. Collectively, our results clearly demonstrated that the protein stability of AXIN1 was regulated by a CK1ε‐SIAH1 axis in CRC cells. This study uncovered a novel mechanism underlying the oncogenic roles of CK1ε in colorectal tumorigenesis and progression.

## Conflict of interest

The authors declare no conflict of interest.

## Author contributions

DL contributed to conceptualization, investigation, formal analysis, supervision, funding acquisition, writing original draft, writing review and editing. XZ contributed to conceptualization, investigation, data curation, supervision, funding acquisition, writing original draft, writing review and editing. MY contributed to conceptualization, investigation, data curation, formal analysis, investigation, writing original draft, writing review and editing. XP, HW, HD, and JN contributed to investigation, data curation. ZS contributed to investigation, data curation, funding acquisition. SL, JS, and QS contributed to funding acquisition, conceptualization.

### Peer review

The peer review history for this article is available at https://www.webofscience.com/api/gateway/wos/peer‐review/10.1002/1878‐0261.13624.

## Supporting information


**Fig. S1.** CK1δ/ε inhibitors increase AXIN1 protein in colorectal cancer cells.
**Fig. S2.** Knockdown of AXIN1 attenuates the effect of CK1ε/δ inhibitors on the growth of colorectal cancer cells.
**Fig. S3.** CSNK1E knockdown does not affect AXIN1 mRNA levels.
**Fig. S4.** SR3029 downregulates the mRNA expression of AXIN2.
**Fig. S5.** CK1δ/ε inhibitor suppresses the ubiquitination of AXIN1.
**Fig. S6.** CK1ε phosphorylates AXIN1.
**Fig. S7.** CK1ε synergizes with SIAH1 to regulate AXIN1 stability.
**Fig. S8.** CK1ε cooperates with SIAH1 to promote the viability of CRC cells and upregulate CYCLIND1 expression.
**Fig. S9.** SR3029 enhances the suppressive effect of Siah1α knockdown on colorectal tumor growth *in vivo*.

## Data Availability

The published article includes all data sets generated/analyzed for this study.
