# Supplementary material for: The CK1ε/SIAH1 axis regulates AXIN1 stability in colorectal cancer cells
Source: Mol Oncol. 2024 Feb 28;18(9):2277–97. doi: 10.1002/1878-0261.13624 (PMC11467792; doi:10.1002/1878-0261.13624)
Supplement: Supplementary file 1 — Fig. S1. CK1δ/ε inhibitors increase AXIN1 protein in colorectal cancer cells. Fig. S2. Knockdown of AXIN1 attenuates the effect of CK1ε/δ inhibitors on the growth of colorectal cancer cells. Fig. S3. CSNK1E knockdown does not affect AXIN1 mRNA levels. Fig. S4. SR3029 downregulates the mRNA expression of AXIN2. Fig. S5. CK1δ/ε inhibitor suppresses the ubiquitination of AXIN1. Fig. S6. CK1ε phosphorylates AXIN1. Fig. S7. CK1ε synergizes with SIAH1 to regulate AXIN1 stability. Fig. S8. CK1ε cooperates with SIAH1 to promote the viability of CRC cells and upregulate CYCLIND1 expression. Fig. S9. SR3029 enhances the suppressive effect of Siah1α knockdown on colorectal tumor growth in vivo. [file MOL2-18-2277-s001.docx]

**The CK1ε/SIAH1 axis regulates AXIN1 stability in colorectal cancer cells**

Running title: The CK1ε/SIAH1 axis promotes AXIN1 degradation

Mengfang Yan^1, 2^, Zijie Su^1, 3^, Xiaoyi Pang^1^, Hanbin Wang^1^, Han Dai^1^, Jiong Ning^1^, Shanshan Liu^1^, Qi Sun^1^, Jiaxing Song^1, 4^, Xibao Zhao^1, *^, Desheng Lu^1, 2, *^

1. Guangdong Provincial Key Laboratory of Regional Immunity and Disease, International Cancer Center, Marshall Laboratory of Biomedical Engineering, Department of Pharmacology, Shenzhen University Medical School, Shenzhen University, Shenzhen, Guangdong, 518055, China.
2. School of Pharmacy, Shenzhen University Medical School, Shenzhen University, Shenzhen, Guangdong, 518055, China.
3. Department of Research, The Affiliated Tumor Hospital of Guangxi Medical University, Nanning, China.
4. Medical Scientific Research Center, Life Sciences Institute, Guangxi Medical University, Nanning, China.

^*^ Corresponding author. Guangdong Provincial Key Laboratory of Regional Immunity and Disease, International Cancer Center, Marshall Laboratory of Biomedical Engineering, Department of Pharmacology, Shenzhen University Medical School, Shenzhen University, Shenzhen, Guangdong, 518055, China. Email addresses: xibaozhao@szu.edu.cn (X, Zhao), delu@szu.edu.cn (D, Lu).


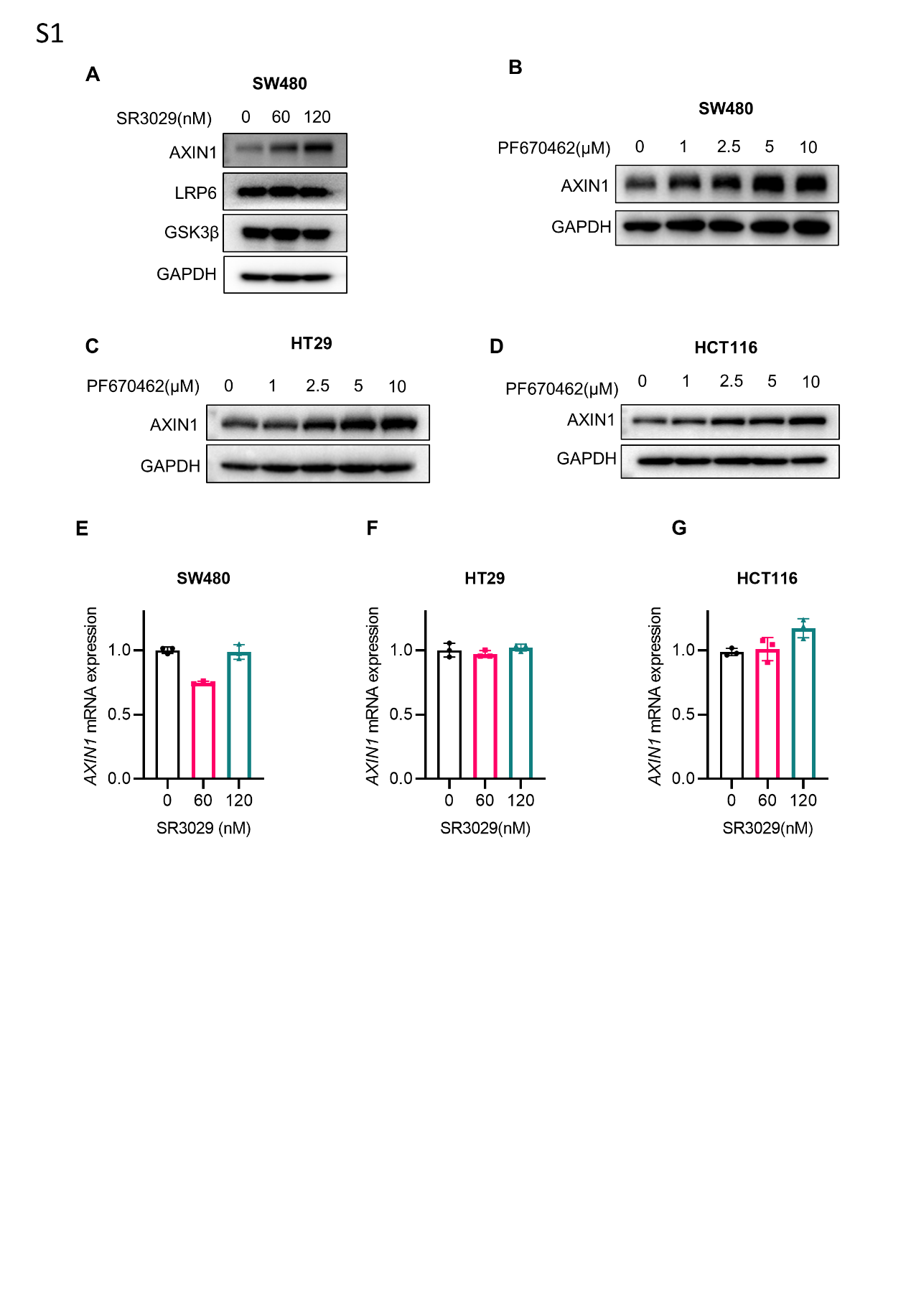


**Supplementary Figure S1 CK1δ/ε inhibitors increase AXIN1 protein in colorectal cancer cells.** (A) Immunoblot analysis of lysates of SW480 cells treated with indicated concentrations SR3029 (0, 60 and 120 nM) for 24 h before harvesting. Shown is one representative of at least three independent experiments. (B-D) Immunoblot analysis of lysates of SW480, HT29 and HCT116 cells treated with indicated concentrations PF670462 (0, 1, 2.5, 5 and 10 μM) for 24 h before harvesting. Shown is one representative of at least three independent experiments. (E-G) SW480, HT29 and HCT116 cells treated with 0, 60 and 120 nM SR3029 for 24 h before harvesting. RT-qPCR was performed to detect the *AXIN1* mRNA expression. Quantification of mRNA level was normalized to *GAPDH* (*n* = 3 independent experiments). Values are shown as means ± SD. Student’s t test.


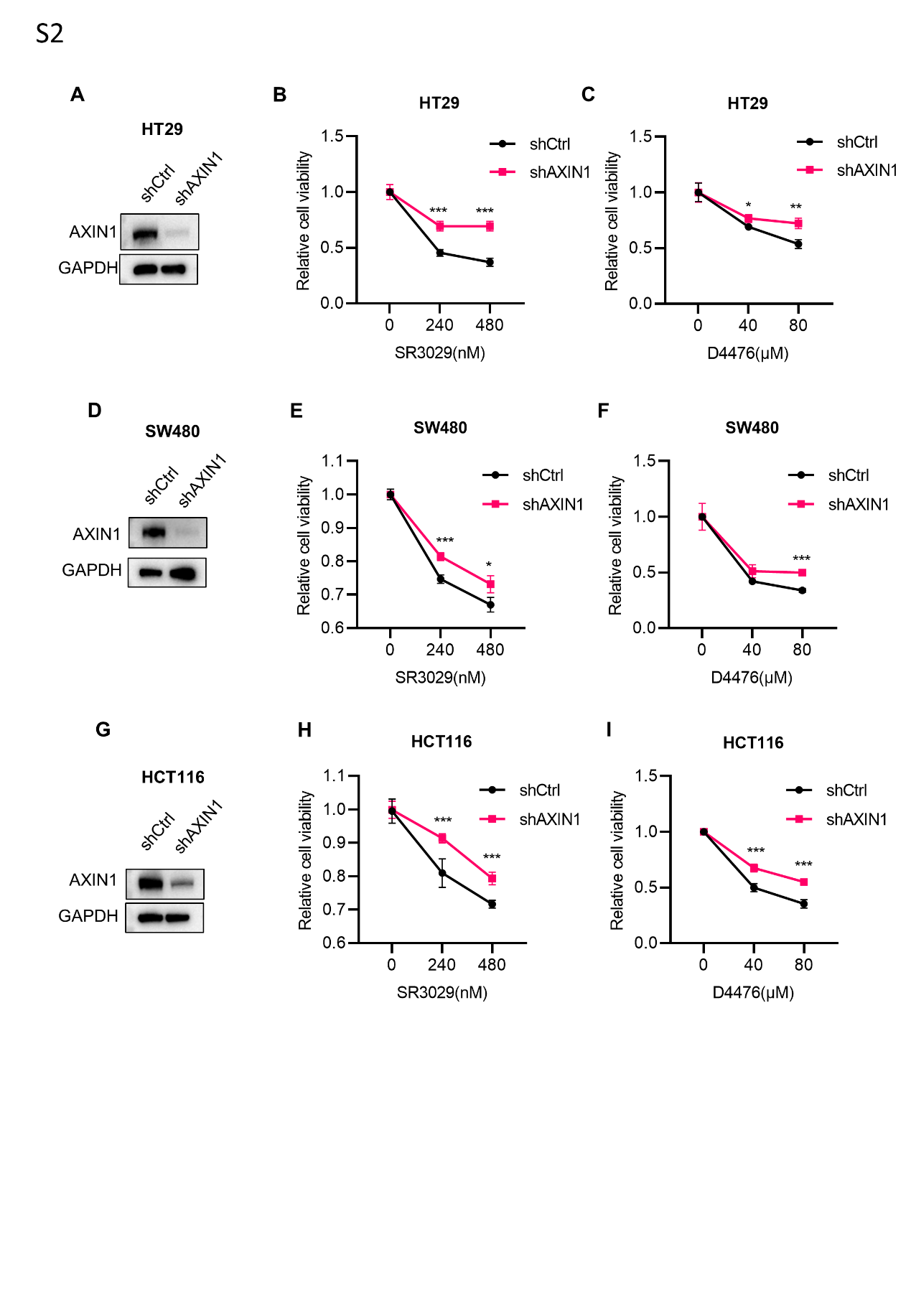


**Supplementary Figure S2 Knockdown of AXIN1 attenuates the effect of CK1ε/δ inhibitors on the growth of colorectal cancer cells.** (A, D and G) Immunoblot analysis of lysates of HT29, SW480 and HCT116 cells infected with shCtrl and shAXIN1 lentivirus. Shown is one representative of at least three independent experiments. (B, E and H) The MTT assay was performed to assess the viability of SW480, HT29, and HCT116 cells after infection with shCtrl or shAXIN1 lentivirus. The cells were treated with varying concentrations of SR3029 (0, 240 and 480 nM) for 24 h. (C, F and I) Similar to panels B, E and H except the cells were treated with varying concentrations of D4476 (0, 40 and 80 μM) for 24 h (*n*=5 independent experiments). Values are shown as means ± SD. *p<0.05, **p<0.01, ***p<0.001; Student’s t test.


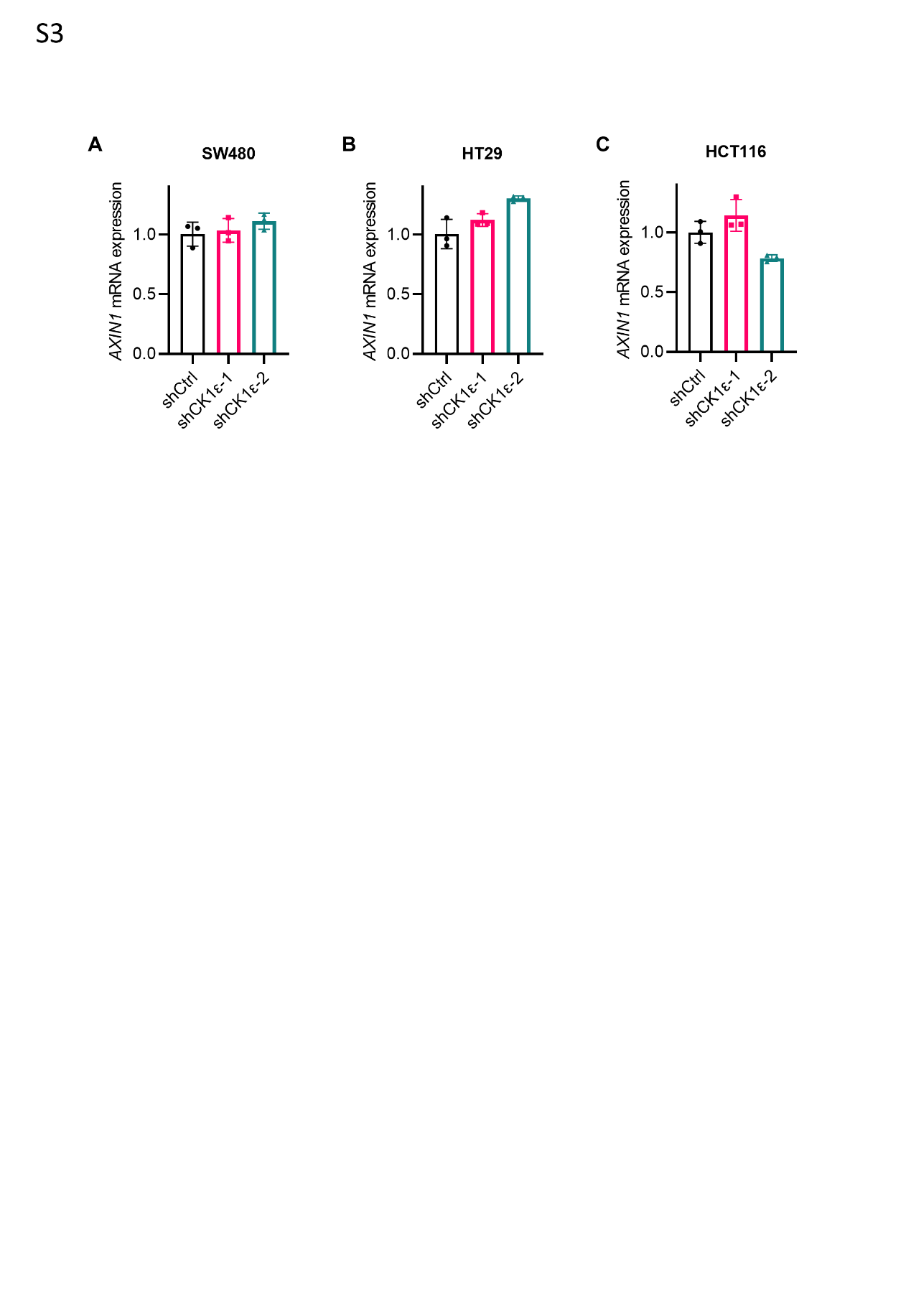


**Supplementary Figure S3 *CSNK1E* knockdown does not affect *AXIN1* mRNA levels.** (A, B and C) SW480, HT29 and HCT116 cells infected with shCtrl, shCK1ε-1 and shCK1ε-2 lentivirus. RT-qPCR was performed to detect the *AXIN1* mRNA expression. Quantification of mRNA level was normalized to *GAPDH* (*n*=3 independent experiments). Values are shown as means ± SD. Student’s t test.


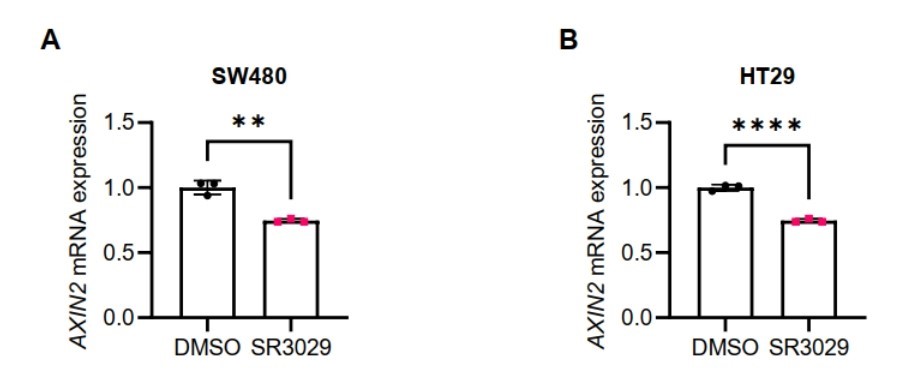


**Supplementary Figure S4 SR3029 downregulates the mRNA expression of *AXIN2*.** (A, B) RT-qPCR was performed to detect the *AXIN2* mRNA expression in SW480 and HT29 cells treated with 100 nM SR3029 for 24 h before harvesting. Quantification of mRNA level was normalized to *GAPDH* (*n*=3 independent experiments). Values are shown as means ± SD. **P<0.01, ***P<0.001; Student’s t test.


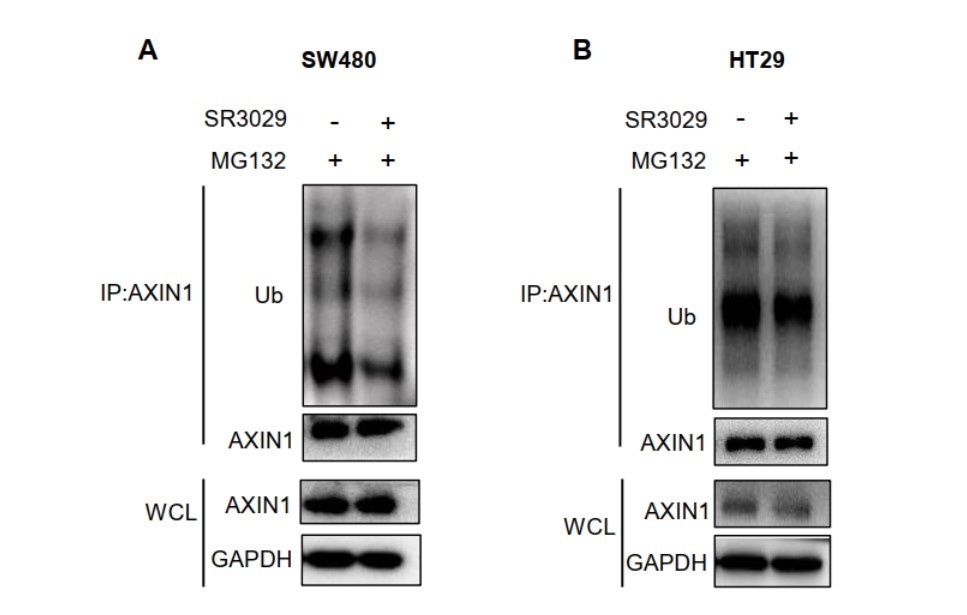


**Supplementary Figure S5 CK1δ/****ε inhibitor suppresses the ubiquitination of AXIN1.** (A-B) Immunoprecipitation and immunoblot analysis of lysates of SW480 and HT29 cells treated with or without 100 nM SR3029 for 24 h and 10 μM MG132 for 8 h before harvesting. Shown is one representative of at least three independent experiments.


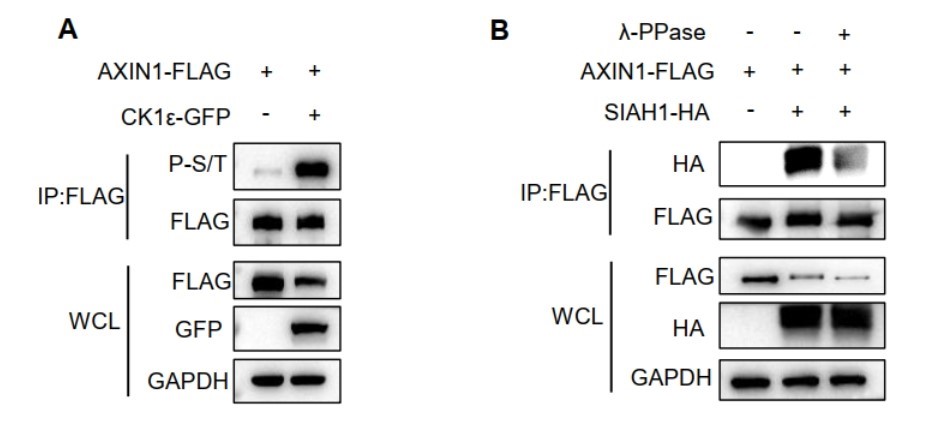


**Supplementary Figure S6 CK1ε phosphorylates AXIN1.** (A) Immunoprecipitation and immunoblot analyses were performed on lysates of HEK293T cells transfected with various plasmids encoding GFP-CK1ε and FLAG-AXIN1. (B) Immunoprecipitation and immunoblot analyses were performed on lysates of HEK293T cells transfected with various plasmids encoding HA-SIAH1 and FLAG-AXIN1. The cells were treated with or without 400 units of λ-PPase before immunoprecipitation with anti-FLAG beads. Shown is one representative of at least three independent experiments.


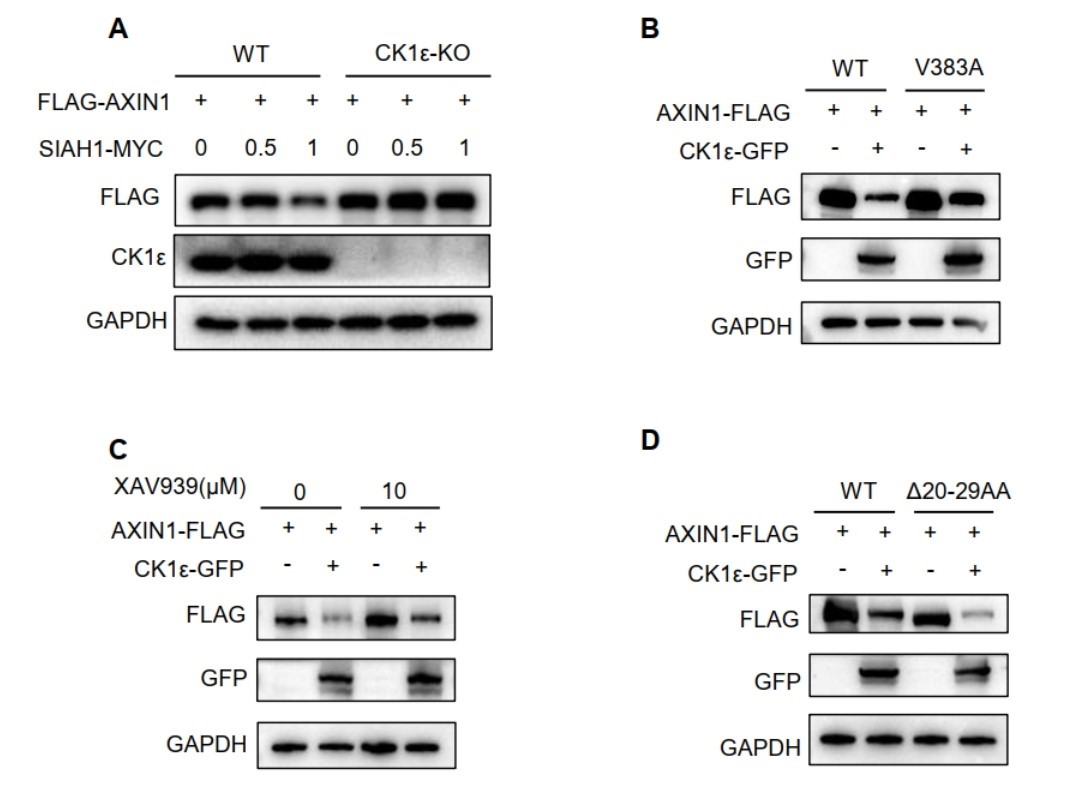


**Supplementary Figure S7 CK1ε synergizes with SIAH1 to regulate AXIN1 stability.** (A) Immunoblot analysis of lysates of *CSNK1E*-deficient or control cells transfected with of indicated concentrations plasmids encoding MYC-tagged SIAH1 and FLAG-tagged AXIN1. (B) Immunoblot analysis of lysates of HEK293T cells transfected with of indicated concentrations plasmids encoding GFP-tagged CK1ε and WT or V383A FLAG-tagged AXIN1. (C) Immunoblot analysis of lysates of HEK293T cells transfected with of indicated concentrations plasmids encoding GFP-tagged CK1ε and FLAG-tagged AXIN1 and then treated with 10 μM XAV939 for 24 h before harvesting. (D) Immunoblot analysis of lysates of HEK293T cells transfected with of indicated concentrations plasmids encoding GFP-tagged CK1ε and WT or △20-29AA FLAG-tagged AXIN1. Shown is one representative of at least three independent experiments.


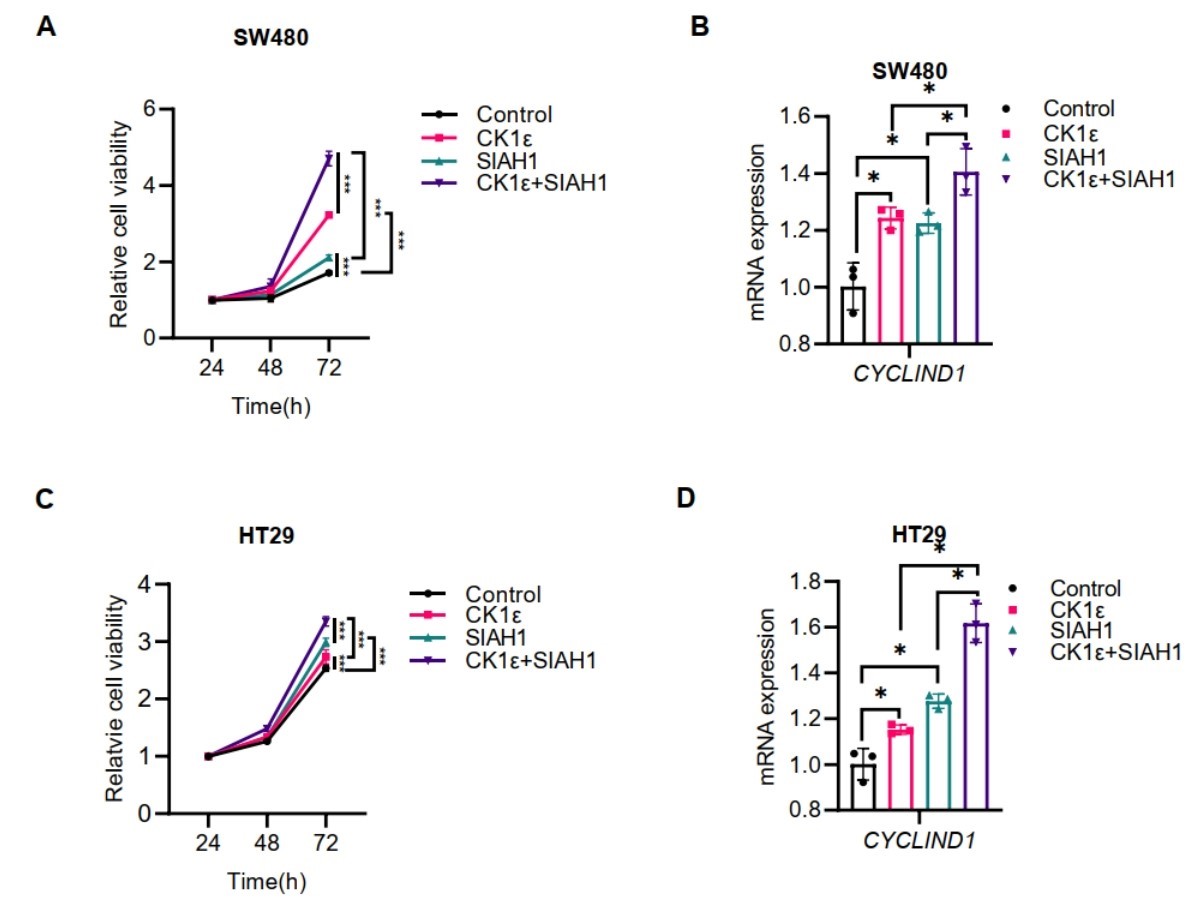


**Supplementary Figure S8 CK1ε cooperates with SIAH1 to promote the viability of CRC cells and upregulate *CYCLIND1* expression.** SW480 and HT29 cells were infected with CK1ε or SIAH1 lentivirus alone, or CK1ε together with SIAH1 lentivirus. (A, C) MTT assay was used to detect cell viability. (B, D) RT-qPCR was performed to detect mRNA expression of the Wnt target gene *CYCLIND1*. Quantification of mRNA level was normalized to *GAPDH* (*n*=3 independent experiments). Values are shown as means ± SD. *P<0.05, **P<0.01, ***P<0.001; Student’s t test.


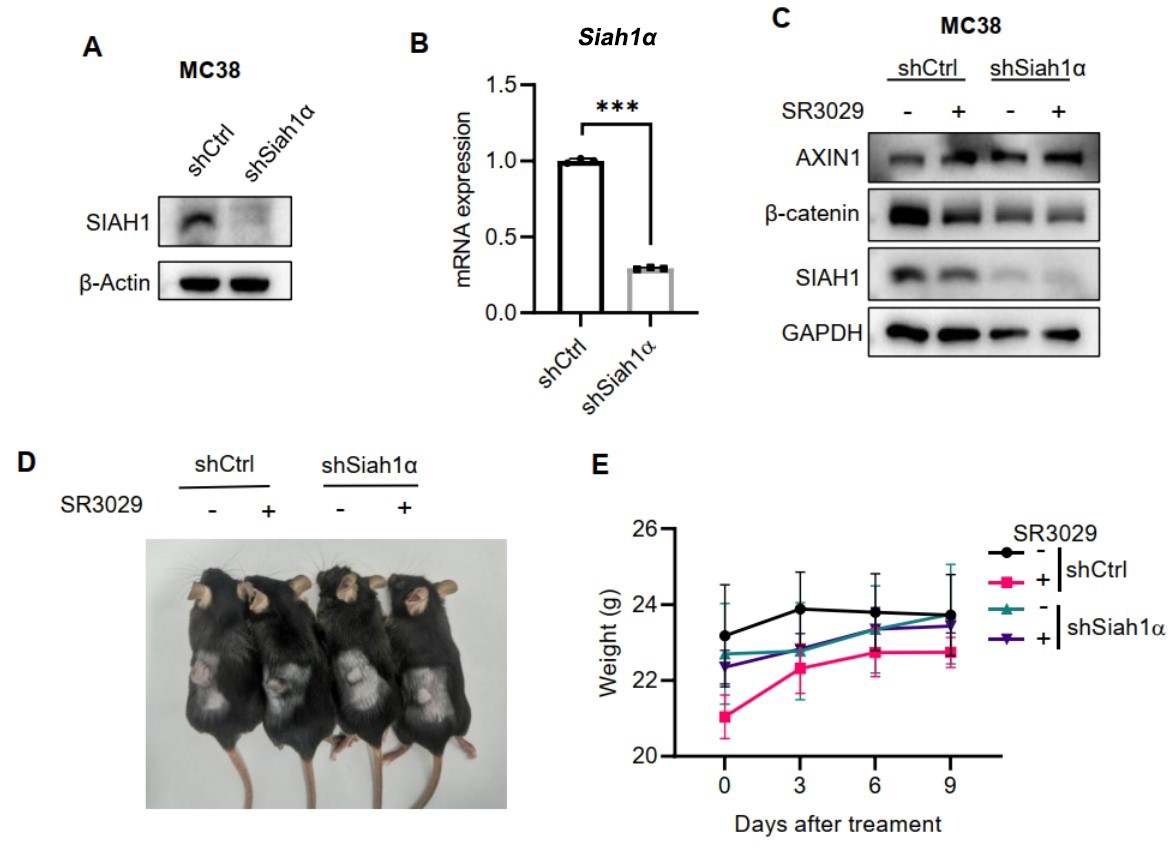


**Supplementary Figure S9 SR3029 enhances the suppressive effect of *Siah1α* knockdown on colorectal tumor growth *in vivo*.** (A) Immunoblot analysis of lysates of MC38 cells infected with shCtrl and shSiah1α lentivirus. (B) RT-qPCR was performed to detect the *Siah1α* mRNA expression of MC38 cells infected with shCtrl and shSiah1α lentivirus. Quantification of mRNA level was normalized to *Gapdh* (*n*=3 independent experiments). (C) Immunoblot analysis of lysates of MC38 cells infected with shCtrl or shSiah1α lentivirus and then treated with 100 nM SR3029 for 24 h before harvesting. (D) Images of represent mice from each experimental group. (E) Mean body weight of each experimental group (*n*=5 independent experiments). Shown is one representative of at least three independent experiments. Values are shown as means ± SD. ***P<0.001; Student’s t test.
